# MPT64 antigen detection test improves routine diagnosis of extrapulmonary tuberculosis in a low-resource setting: A study from the tertiary care hospital in Zanzibar

**DOI:** 10.1371/journal.pone.0196723

**Published:** 2018-05-09

**Authors:** Melissa Davidsen Jørstad, Msafiri Marijani, Anne Ma Dyrhol-Riise, Lisbet Sviland, Tehmina Mustafa

**Affiliations:** 1 Department of Thoracic Medicine, Haukeland University Hospital, Bergen, Norway; 2 Centre for International Health, Department of Global Public Health and Primary Care, University of Bergen, Bergen, Norway; 3 Department of Diagnostic Services, Mnazi Mmoja Hospital, Zanzibar, The United Republic of Tanzania; 4 Department of Clinical Science, Faculty of Medicine, University of Bergen, Bergen, Norway; 5 Department of Infectious Diseases, Oslo University Hospital, Oslo, Norway; 6 Institute of Clinical Medicine, Faculty of Medicine, University of Oslo, Oslo, Norway; 7 Department of Clinical Medicine, Faculty of Medicine, University of Bergen, Bergen, Norway; 8 Department of Pathology, Haukeland University Hospital, Bergen, Norway; Hospital Universitari de Bellvitge, SPAIN

## Abstract

**Background:**

Extrapulmonary tuberculosis (EPTB) is a diagnostic challenge. An immunochemistry-based MPT64 antigen detection test (MPT64 test) has reported higher sensitivity in the diagnosis of EPTB compared with conventional methods. The objective of this study was to implement and evaluate the MPT64 test in routine diagnostics in a low-resource setting.

**Methods:**

Patients with presumptive EPTB were prospectively enrolled at Mnazi Mmoja Hospital, Zanzibar, and followed to the end of treatment. Specimens collected were subjected to routine diagnostics, GeneXpert® MTB/RIF assay and the MPT64 test. The performance of the MPT64 test was assessed using a composite reference standard, defining the patients as tuberculosis (TB) cases or non-TB cases.

**Results:**

Patients (n = 132) were classified as confirmed TB (n = 12), probable TB (n = 34), possible TB (n = 18), non-TB (n = 62) and uncategorized (n = 6) cases. Overall, in comparison to the composite reference standard for diagnosis, the sensitivity, specificity, positive predictive value, negative predictive value and accuracy of the MPT64 test was 69%, 95%, 94%, 75% and 82%, respectively. The MPT64 test performance was best in TB lymphadenitis cases (n = 67, sensitivity 79%, specificity 97%) and in paediatric TB (n = 41, sensitivity 100%, specificity 96%).

**Conclusions:**

We show that the MPT64 test can be implemented in routine diagnostics in a low-resource setting and improves the diagnosis of EPTB, especially in TB lymphadenitis and in children.

## Introduction

Despite efforts to develop new diagnostic tools for tuberculosis (TB), the diagnosis of extrapulmonary TB (EPTB) remains a challenge. The various clinical presentations of EPTB are non-specific, and the disease is often paucibacillary leading to low sensitivities of routine diagnostic methods such as; acid-fast bacilli (AFB) microscopy [1–3] and culture [1, 4, 5]. Furthermore, mycobacterial culture has a long turnaround time, and its technical and logistic demands limits its use in resource-limited settings. Histology can be used in the diagnosis of EPTB, but lacks specificity as several other conditions may present similar histological features [6]. Most nucleic acid amplification tests show better sensitivity, but are complex, expensive, technically demanding and prone to contamination, limiting their use in low-resource diagnostic settings [7–10]. The development of the GeneXpert® MTB/RIF (Xpert) assay is a landmark in TB diagnostics, but reported sensitivities of the assay for EPTB samples are highly heterogeneous and vary widely across different sample types [11–14]. Due to lack of a low-cost, robust, rapid and accurate diagnostic method, EPTB is either over- or underdiagnosed, leading to increased morbidity and mortality. Thus, there is a need for better diagnostic tools, which are implementable and sustainable in resource-limited settings.

MPT64 is a protein secreted by the *Mycobacterium tuberculosis* (Mtb) complex species, not detected in non-tuberculous mycobacteria (NTM) [15, 16] and bacillus Calmette-Guérin strains with RD2 deletion [17]. Earlier studies have investigated the diagnostic potential of an immunochemistry-based MPT64 antigen detection test (MPT64 test) showing sensitivity and specificity comparable to nested polymerase chain reaction (PCR) [4, 5, 18, 19].

Zanzibar is a semi-autonomous region of the United Republic of Tanzania and comprises the main islands Unguja and Pemba. The region has 1.3 million inhabitants [20], a prevalence of bacteriologically confirmed pulmonary TB of 124 per 100 000 [21], and a low adult human immunodeficiency virus (HIV) prevalence of 1% [22]. In 2013, 30% of the new TB patients were registered as EPTB cases [23]. The aim of the present study was to implement and evaluate the performance of the MPT64 test in routine diagnostics at the tertiary care hospital in Zanzibar, a low-resource setting with a high TB burden.

## Materials and methods

### Study participants

The study was conducted at Mnazi Mmoja Hospital (MMH), Unguja, Zanzibar. MMH is the only tertiary referral hospital in Zanzibar, and provides in addition primary and secondary health care for some districts. Patients of all ages presenting with symptoms suggestive of EPTB were prospectively enrolled from hospital wards and out-patients departments between 1^st^ August 2014 and 31^st^ August 2015. Patients who consented and where a representative sample was collected were included in the study. Those who had received anti-TB treatment (ATT) during the previous year were excluded. All patients were interviewed using a pretested structured questionnaire, and a physical examination was performed. Diagnostic imaging was done if required and possible. Response to ATT was assessed at 2–3 months and at the end of treatment by using criteria based on improvement in signs and symptoms, weight gain and objective measures such as repeated chest radiographs, abdominal ultrasound and reduction of lymph node swellings. Patients not starting ATT were followed until recovery or until a diagnosis other than TB was established.

### Study questionnaire

The study questionnaire was developed in English, translated to Swahili, then translated back to English. The translations were performed by two separate individuals fluent in both languages. The original English version and the back-translated version were compared to assess the validity. Prior to testing of the questionnaire among patients, two bilingual individuals at Zanzibar evaluated both the English (S1 and S2 Texts) and Swahili versions (S3 and S4 Texts) to assess the meaning of the questions according to the local setting. The questionnaire was tested among three adult inpatients at the medical ward at MMH to identify unclear or ambiguous questions and the questionnaire was adjusted accordingly.

### Sample collection and processing

Fine-needle aspiration cytology (FNAC) from peripheral lymph nodes was performed by the hospital pathologist (MM) using a 23-g needle. Four smears were prepared from each aspirate; one each for cytology and AFB microscopy, and two for immunocytochemical (ICC) staining. The slides for ICC staining were fixed in 95% alcohol before being transported to the laboratory. The needle was rinsed with 2 ml of sterile 0.9% saline solution and distributed equally for the Xpert assay and Mtb culture. All fluids were aspirated aseptically, and subjected to routine diagnostic investigations, in addition to the Xpert assay. The specimens were centrifuged at 3000g for 10 minutes and smears were made from the 20μl of the sediment for cytology, AFB microscopy and ICC staining. The biopsies were divided equally and one half transported in 0.9% saline for Mtb culture and the other half fixed in 4% phosphate buffered formaldehyde for conventional paraffin embedding. From the formalin-fixed, paraffin-embedded biopsies, five-μm-thick tissue sections were prepared for histology, AFB microscopy and immunohistochemical (IHC) staining.

### Diagnostic procedure

AFB microscopy was performed using Ziehl-Neelsen (ZN) staining. Culture was done at the Public Health Laboratory–Ivo de Carneri (PHL-IdC) located at Pemba island, on Lowenstein-Jensen medium according to the standard protocol. Positive cultures were confirmed by smear microscopy and sent to the Central Tuberculosis and Leprosy Reference Laboratory at Tanzania mainland for species identification and drug sensitivity testing. The Xpert assay was performed according to the standard protocol recommended by WHO [24].The specimens were stored at 4°C for a maximum of 7 days if it was not analyzed on the same day as the sampling. The Xpert assay was not performed on biopsies. The slides for cytological and histological examination were stained with Papanicolaou stain and haematoxylin-eosin, respectively. Two laboratory technologists working at MMH were trained to perform the ICC/IHC staining (immunostaining) procedures and the pathologist at MMH (MM) received training in evaluation of the immunostaining. The immunostaining was performed as described earlier [5, 18] with some modifications, by using an in-house polyclonal anti-MPT64 primary antibody at 1/250 dilution and Dako kit (Dako Envision® + System-HRP, K4009, Dako, Glostrup, Denmark), to demonstrate the presence of MPT64 antigens. Briefly, for ICC staining, the slides were hydrated through decreasing grades of alcohol, washed in distilled water for 10 minutes and incubated with hydrogen peroxide for 15 minutes to inhibit the endogenous peroxidase activity. Thereafter, the primary antibody was applied and incubated for 60 minutes. Anti-rabbit dextran polymer conjugated to horseradish peroxidase was then applied to the slides for 45 minutes. To visualize the bound antibody, the slides were incubated for 10 minutes with 3-amino-9-ethylcarbazol and hydrogen peroxide-containing substrate, and the background counterstained with Mayer’s hematoxylin. The slides were mounted in Immu-Mount (Thermo Fisher Scientific). Between the incubation steps the slides were washed with wash buffer (Dako Wash buffer 10x, S3006, Dako, Glostrup, Denmark). For IHC staining, tissue sections were deparaffinized with xylene, hydrated and after microwave antigen retrieval using citrate buffer, pH 6.2, subsequently incubated with hydrogen peroxide for 10 minutes. Additional steps were as in the ICC staining procedure.

### Evaluation of immunostaining

The stained slides were evaluated at 20x magnification using a light microscope, and possible positive signals were further assessed at 40x magnification. The pathologist (MM) evaluating the slides was blinded for the ZN staining and the Xpert assay results. Signals were regarded as positive if seen as reddish granular intracytoplasmic staining or extracellular staining in necrotic areas. The sample was evaluated as weakly positive if 1–2 strong positive or 3 weakly positive spots were seen, as positive if > 2 strong positive spots or > 3 weakly positive spots, negative if no positive signal and as inconclusive if ≤ 2 weakly positive spots or only uncertain spots were seen.

### Patient categories and morphological criteria

The patients were categorized by using a composite reference standard (CRS) combining the various diagnostic criteria into 5 separate groups as described in Table 1. The MPT64 test results were not available during the categorization of patients. Briefly, the morphological criteria taken to be consistent with TB were the presence of granuloma with or without necrosis, poorly formed granulomas with necrosis or necrosis without granulomas in the biopsy specimens. In FNAC smears from lymph nodes these were granulomatous inflammation with or without necrosis or necrotic material without granulomas, and in cytological smears from effusion/cerebrospinal fluid (CSF) the predominance of lymphocytes was taken to be suggestive of tuberculosis.

**Table 1:** Criteria for categorization of patients into various categories of the composite reference standard.

**Confirmed TB case**	Positive mycobacterial culture and/or *M*. *tuberculosis* detected by the Xpert assay
**Probable TB case**	Clinical presumptive EPTB and a good response to ATT at 2–3 months and/or end of treatment **or** clinical presumptive EPTB and bacteriologically confirmed concomitant pulmonary TB **and one of the following** • AFB seen on ZN staining of extrapulmonary material • Radiological findings suggestive of EPTB • Effusions/CSF: lymphocytosis on fluid cytology and protein level > 3 g/dl (> 1 g/l for CSF) • FNAC/biopsy–morphological features consistent with TB
**Possible TB case**	a) Patient started ATT based on clinical presumptive EPTB^a^ **and one of the following** • AFB seen on ZN staining of extrapulmonary material • Radiological findings suggestive of EPTB • Effusions/CSF: lymphocytosis on fluid cytology and protein level > 3 g/dl (> 1 g/l for CSF) • FNAC/biopsy–morphological features consistent with TB b) Clinical presumptive EPTB and a good response to ATT at 2–3 months and/or end of treatment
**Non-TB case**^**b**^ **(control subject)**	Negative mycobacterial culture **and/or** *M*. *tuberculosis* not detected by the Xpert assay **and one of the following** • Improvement without ATT and/or response to specific non-tuberculous therapy • Cytology/histology examination concluded other diagnosis than TB • Alternative diagnosis concluded by the clinician • Patient started on ATT based on clinical presumptive EPTB, but did not respond to treatment
**Uncategorized patient**	not possible to categorize the patient

**NOTE.** TB, tuberculosis; EPTB, extrapulmonary tuberculosis; ATT, antituberculous treatment; AFB, acid fast bacilli; ZN, Ziehl-Neelsen; CSF, cerebrospinal fluid; FNAC, fine-needle aspiration cytology.

^a^ Patient died before observation time to assess response to treatment or was lost to follow-up.

^b^ Culture was missing in 4 cases and the Xpert assay was missing in 28 cases. Among these, 3 patients had neither culture or Xpert assay results.

### Statistical analysis

Data was analyzed using Statistical Package for the Social Sciences (SPSS) for Windows version 24.0. Chi-square test was used to compare differences in categorical variables. The performance of the different diagnostic procedures was calculated using the CRS as a reference. Cross-tabulation was used to calculate sensitivity, specificity, positive predictive value (PPV), negative predictive value (NPV) and accuracy. *P* value < 0.05 was considered statistically significant.

### Ethical considerations

Ethical clearance was obtained from the Regional Committee for Medical and Health Research Ethics, Western-Norway (REK Vest) and the Zanzibar Medical Research and Ethics Committee (ZAMREC). All study participants provided informed written consent. For children, consent was provided by the parent/guardian, in addition, children between 7–18 years had to sign the consent form as well. The biological specimens were collected on clinical demand and not based on participation in the study.

## Results

**Fig 1:**
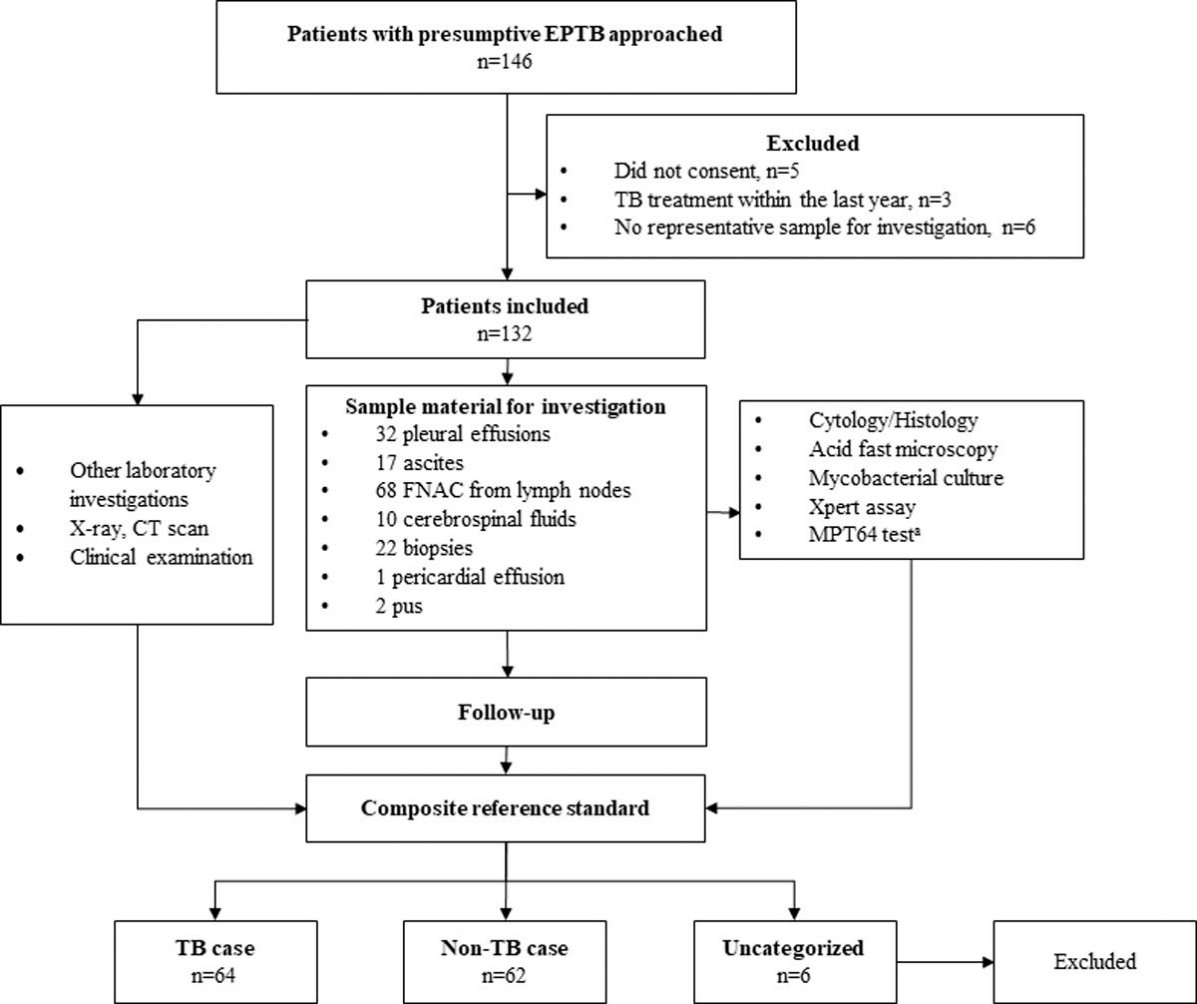
Flow-chart showing the study design and patient flow in the study. **NOTE.** EPTB, extrapulmonary tuberculosis; TB, tuberculosis; CT, computed tomography; FNAC, fine-needle aspiration cytology. ^a^ Not included in the composite reference standard.

A total of 146 patients were approached and 132 patients were enrolled in the study. The total number of collected biological specimens were 152 from the 132 study participants. Fig 1 provides an overview of patients included and specimens collected in the study. According to the CRS, 12 (9%) were categorized as confirmed TB cases; 34 (26%) as probable TB cases; 18 (14%) as possible TB cases, 62 (47%) as non-TB cases and 6 (5%) patients were uncategorized. The uncategorized patients and the specimens collected in these patients were excluded from further analyses. Thus, 126 patients and the laboratory results from 145 specimens were included in the data analysis. In most patients, one specimen from the presumptive site of infection was collected and examined with the various diagnostic procedures. Two different specimens were collected from the same site in 19 patients (FNAC and biopsy, n = 17; ascites and biopsy, n = 1; pericardial effusion and biopsy, n = 1). All specimens were examined with the MPT64 test, whereas the routine methods were missed in some specimens; 143 (99%), 125 (86%) and 72 (50%) of the specimens were examined with ZN staining, culture and the Xpert assay, respectively.

### Clinical characteristics

The demographic and baseline characteristics, as well as the distribution of presumptive sites of infection among the study participants, are described in Table 2. The age distribution differed significantly between the TB and non-TB cases. The majority of the TB patients were between 15–44 years (59%), whereas the non-TB patients were predominantly either children (40%) or above 44 years (29%). HIV status was known in 94 patients, and 21% of these were HIV positive. In the HIV positive patients, 14/20 (70%) were categorized as TB cases; 3 as confirmed TB, 5 as probable TB and 6 as possible TB cases, respectively. Overall, there was a significant difference in the presumptive sites of EPTB between adults and children (*P* = .047). In children, there was a higher proportion of lymphadenitis 29/41 (71%), and lower proportions of pleuritis 7/41 (17%), peritonitis 2/41 (5%) and other sites 3/41 (7%), while the corresponding proportions among adults were 38/85 (45%), 24/85 (28%), 14/85 (17%) and 9/85 (11%), respectively. Among the paediatric TB cases (n = 16) the sites of infection were TB lymphadenitis (n = 11), pleural TB (n = 3), abdominal TB (n = 1) and TB pericarditis (n = 1).

**Table 2:** Demographic and baseline characteristics of the 126 categorized study participants, n(%).

Characteristics	TB cases^a^ n = 64	Non-TB cases n = 62	*P* value^b^
**Sex**			.842
Male	35 (55)	35 (56)	
Female	29 (45)	27 (44)	
**Age (years)**			.014*
< 15	16 (25)	25 (40)	
15–29	17 (27)	8 (13)	
30–44	21 (33)	11 (18)	
≥45	10 (16)	18 (29)	
**In/outpatient**			.216
Inpatient	22 (34)	28 (45)	
Outpatient	42 (66)	34 (55)	
**HIV status**			.517^c^
Positive	14 (23)	6 (18)	
Negative	46 (77)	28 (82)	
Unknown	4 (-)	28 (-)	
**Presumptive site of infection**			.177
Lymphadenitis	34 (53)	33 (53)	
Pleuritis	20 (31)	11 (18)	
Peritonitis	6 (9)	10 (16)	
Other sites^d^	4 (6)	8 (13)	
**Symptoms/signs at time of inclusion**			.189
Local	14 (22)	20 (32)	
Local and systemic	50 (78)	42 (68)	

**NOTE**. TB, tuberculosis; HIV, human immunodeficiency virus.

^a^ Confirmed, probable and possible TB cases.

^b^ Comparing group differences between TB and non-TB cases.

^c^ Only comparing patients with known HIV status.

^d^ TB cases, meningitis (n = 2), spondylitis (n = 1), pericarditis (n = 1); Non-TB cases, meningitis (n = 6), osteomyelitis (n = 1), mastitis (n = 1).

* Statistically significant.

Most patients (73%) presented with both local and systemic signs and symptoms, more so in the TB cases compared to non-TB cases, but the difference in proportions was not significant. The final diagnoses among the non-TB cases were malignant tumor (n = 18), benign tumor (n = 5), benign reactive lymphadenopathy (n = 13), heart failure (n = 5), liver disease (n = 6), meningitis/encephalitis (n = 6), pneumonia (n = 1), endometriosis (n = 1), hydatid cyst (n = 1), sialadenitis (n = 1) and sclerosing lymphocytic mastitis (n = 1). In 1 patient spontaneous resolution of ascites and pleural effusion was observed, 2 patients did not respond to anti-TB treatment and malignancy was suspected but not confirmed, and in 1 patient the treating physician did not suspect TB after throughout evaluation.

### MPT64 test performance compared to routine laboratory diagnostic tests and the Xpert assay

**Fig 2:**
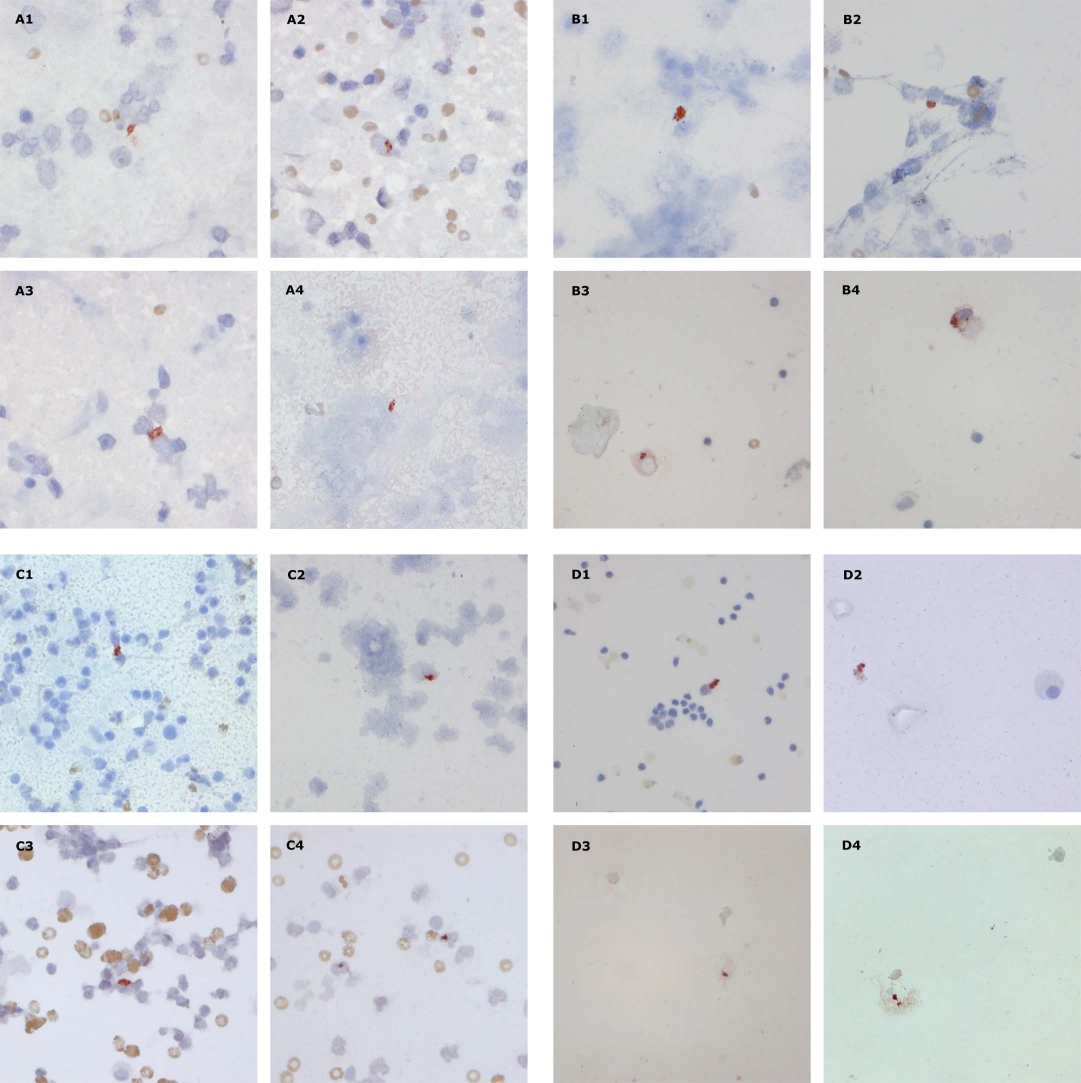
Patterns of immunostaining with anti-MPT64 antibody in various specimens. The signals are seen as granular, reddish staining. A, fine-needle aspirates from lymph nodes, signals were extracellular probably due to cell lysis (A1), mostly intracytoplasmic (A2-A3), and in necrotic areas (A4); B, pleural effusion, intracytoplasmic staining; C1-C2, pus/abscess, intracytoplasmic staining; C3-C4, pericardial effusion, intracytoplasmic staining, and non-specific staining mainly of red blood cells; D1-D2, ascites, intracytoplasmic staining (D1), extracellular probably due to cell lysis (D2); D3-D4, cerebrospinal fluid, extracellular probably due to cell lysis.

The results of all diagnostic procedures among various categories of patients and from available specimens are presented in Table 3. The MPT64 test was positive in a higher proportion of specimens as compared to the other tests. In total, 65% of the specimens in TB cases demonstrated a positive MPT64 test, compared to 12%, 13% and 16% demonstrating positive results with ZN staining, culture and the Xpert assay, respectively. In specimens examined with all diagnostic tests, the MPT64 test was positive in 81%, compared to 14%, 16% and 16% of the specimens showing a positive result with ZN staining, culture and the Xpert assay, respectively (Table 3). In confirmed TB cases 83% of the specimens had a positive MPT64 test as compared to 67% positivity for culture and the Xpert assay. All ZN and/or Xpert assay positive samples were positive with the MPT64 test. Among culture positive samples, 6/8 (75%) were positive with the MPT64 test. FNAC from lymph nodes was the specimen with the highest number of positive MPT64 results (76%) compared to pleural fluid, ascites and CSF. Further, all FNAC from lymph nodes that were positive by ZN staining, culture and/or the Xpert assay were also positive with the MPT64 test. In non-TB cases, the MPT64 test was negative in 73/76 (96%) of the specimens. Fig 2 shows the staining pattern at various sites of infection.

**Table 3:** Results of diagnostic procedures in effusions, CSF, aspirates and biopsies.

Final diagnosis	Total number of specimens	Number of specimens (%) positive by			
		ZN	LJ culture	Xpert assay	MPT64 test
**TB cases–all tests performed**^**a**^					
All samples	37	5/37 (14)	6/37 (16)	6/37 (16)	30/37 (81)
FNAC LN	21	4/21 (19)	4/21 (19)	6/21 (29)	19/21 (90)
Pleural effusion	8	0/8 (-)	0/8 (-)	0/8 (-)	5/8 (63)
**TB cases**^**b**^					
All samples	69	8/69 (12)	8/60 (13) ^c^	6/38 (16) ^d^	45/69 (65)
FNAC LN	34	6/34 (18)	5/30 (17)	6/22 (27)	26/34 (76)
Pleural effusion	20	0/20 (0)	1/19 (5)	0/8 (0)	10/20 (50)
Ascites	6	0/6 (0)	1/6 (17)	0/5 (0)	4/6 (67)
CSF	2	0/2 (0)	1/1 (100)	0/1 (0)	1/2 (50)
Biopsies	5	1/5 (20)	0/2 (0)	-	2/5 (40)
Pericardial effusion	1	0/1 (0)	0/1 (0)	0/1 (0)	1/1 (100)
Pus	1	1/1 (100)	0/1 (0)	0/1 (0)	1/1 (100)
**Confirmed TB cases**	12	3/12 (25)	8/12 (67)	6/9 (67)	10/12 (83)
**Probable TB cases**	39	5/39 (13)	0/31 (0)	0/18 (0)	20/39 (51)
**Possible TB cases**	18	0/18 (0)	0/17 (0)	0/11 (0)	15/18 (83)
**Non-TB cases**^**e**^					
All samples	76	0/74 (0) ^f^	0/65 (0) ^g^	0/34 (0) ^h^	3/76 (4)
FNAC LN	32	0/31 (0)	0/31 (0)	0/20 (0)	0/32 (0)
Pleural effusion	11	0/11 (0)	0/10 (0)	0/5 (0)	1/11 (9)
Ascites	10	0/10 (0)	0/9 (0)	0/3 (0)	1/10 (10)
CSF	6	0/6 (0)	0/6 (0)	0/6 (0)	0/6 (0)
Biopsies	16	0/15 (0)	0/8 (0)	-	1/16 (6)
Pericardial effusion	0	-	-	-	-
Pus	1	0/1 (0)	0/1 (0)	-	0/1 (0)

**NOTE.** CSF, cerebrospinal fluid; ZN, Ziehl-Neelsen staining; LJ, Lowenstein-Jensen; TB, tuberculosis; FNAC, fine-needle aspiration cytology; LN, lymph node.

^a^ Only specimens analyzed with all methods (ZN, LJ culture, Xpert and MPT64 test).

^b^ Five patients with two different specimens from the same site (FNAC and biopsy (n = 4), pericardial effusion and biopsy (n = 1)).

^c^ Contaminated (n = 2) and specimens not sent for culture (n = 7) excluded.

^d ^Invalid results (n = 1) and specimens not analyzed with the Xpert assay (n = 30) excluded.

^e^ Fourteen patients with two different specimens from same site (FNAC and biopsy (n = 13), ascites and biopsy (n = 1)).

^f ^Specimens not examined with ZN (n = 2) excluded.

^g^ Contaminated (n = 1) and specimens not sent for culture (n = 10) excluded.

^h^ Specimens not analyzed with the Xpert assay (n = 42) excluded.

### Diagnostic validation of the MPT64 test

The diagnostic validity of the MPT64 test and other methods in lymphadenitis, pleuritis and paediatric TB using the CRS as reference standard are shown in Table 4. The sensitivity, NPV and accuracy of the MPT64 test was better than the other diagnostic tests. The performance of the MPT64 test was best in TB lymphadenitis, were the sensitivity of the MPT64 test was significantly higher as compared to TB pleuritis (*P* = .025).

**Table 4:** Diagnostic validation of various procedures among lymphadenitis, pleuritis and children using the CRS as reference standard.

	Number of patients	Sensitivity % (95% CI)	Specificity % (95% CI)	PPV %	NPV %	Accuracy %
**Lymphadenitis**^a^	67					
MPT64 test	67	79 (62–91)	97 (84–100)	96	82	88
ZN	67	18 (7–35)	100 (89–100)	100	54	58
Culture	62	16 (5–34)	100 (89–100)	100	54	58
Xpert assay	42	27 (11–50)	100 (83–100)	100	56	62
**Pleuritis**	31					
MPT64 test	31	50 (27–73)	91 (59–100)	91	50	65
ZN	31	0 (0–17)	100 (72–100)	NA	35	35
Culture	29	5 (0–26)	100 (69–100)	100	36	38
Xpert assay	13	0 (0–37)	100 (48–100)	NA	38	38
**Children**	41					
MPT64 test	41	100 (79–100)	96 (80–100)	94	100	98
ZN	41	13 (2–38)	100 (86–100)	100	64	66
Culture	38	19 (4–46)	100 (85–100)	100	63	66
Xpert assay	22	10 (0–45)	100 (74–100)	100	57	59

**NOTE.** CRS, composite reference standard; PPV, positive predictive value; NPV, negative predictive value; CI, confidence interval; ZN, Ziehl-Neelsen staining; NA, not applicable.

^a^ Results from FNAC and biopsy (n = 17) are combined.

The performance of the MPT64 test was better in children (n = 41) as compared to adults (n = 85) with a sensitivity of 100% and 58% (*P* = .002) and a specificity of 96% and 95%, respectively. In the HIV positive patients (n = 20) the sensitivity of the MPT64 test was lower compared to HIV negative cases (n = 74) (57% and 70%, respectively), but the difference was not significant.

### Cytology/histology

In FNAC from lymph nodes, cytomorphological features consistent with TB were reported in only 19/34 of the cases, even though the majority of these patients (78%) were HIV negative. The proportion of HIV positives was slightly lower among the cases with cytology consistent with TB (16%) as compared to those without (31%), but the difference was not statistically significant. The sensitivity of cytology to detect TB was thus 56%. Table 5 shows the results of the various diagnostic procedures in relation to the cytomorphological features. The MPT64 test was positive in 14/19 (74%) of the cases showing cytomorphological patterns consistent with TB, while ZN staining, culture and the Xpert assay were positive in only 5/19 (26%), 4/15 (27%) and 4/12 (33%), respectively. Among the TB patients without cytomorphological features consistent with TB, the MPT64 test was positive in 12/15 (80%), ZN staining in 1/15 (7%), culture in 1/15 (7%) and the Xpert assay in 2/10 (20%) of the patients. In 4/34 TB lymphadenitis cases a lymph node biopsy was performed and the histomorphological picture showed granulomatous inflammation with necrosis (n = 3) and necrosis without granulomas (n = 1). In these biopsies, the MPT64 test was positive in 1/4 (25%). Biopsy of pericardium was performed in one TB patient showing necrosis infiltrated by inflammatory cells, the MPT64 test gave a positive result in this biopsy.

**Table 5:** Relationship between various cytomorphological features in fine-needle aspirates from lymph nodes and results of diagnostic procedures.

	Number of specimens positive (%) by			
Cytomorphology	ZN	Culture	Xpert assay	MPT64 test
**TB cases (n = 34)**				
Gr. infl with necrosis (n = 3) *	0/3 (0)	0/2 (0)	0/1 (0)	1/3 (33)
Gr. infl without necrosis (n = 1) *	0/1 (0)	0/1 (0)	0/1 (0)	1/1 (100)
Supp. infl with necrosis (n = 6) *	1/6 (17)	2/5 (40)	2/4 (50)	6/6 (100)
Lymphoid cells and necrosis (n = 4) *	2/4 (50)	1/3 (33)	1/2 (50)	3/4 (75)
Abundant necrosis (n = 5) *	2/5 (40)	1/4 (25)	1/4 (25)	3/5 (60)
Reactive lymph node hyperplasia (n = 5) **	0/5 (0)	0/5 (0)	0/3 (0)	5/5 (100)
Acute supp. infl (n = 8) **	1/8 (13)	1/8 (13)	2/7 (29)	7/8 (88)
Inconclusive (n = 2) **	0/2 (0)	0/2 (0)	-	0/2 (0)
**Non-TB cases (n = 32)**				
Reactive lymph node hyperplasia (n = 13)	0/13 (0)	0/12 (0)	0/8 (0)	0/13 (0)
Acute supp. infl (n = 2)	0/2 (0)	0/2 (0)	0/1 (0)	0/2 (0)
Abundant necrosis (n = 1)	0/1 (0)	0/1 (0)	-	0/1 (0)
Benign tumor (n = 3)	0/3 (0)	0/3 (0)	0/1 (0)	0/3 (0)
Malign tumor (n = 10)	0/9 (0)	0/10)	0/9 (0)	0/10 (0)
Inconclusive (n = 3)	0/3 (0)	0/3(0)	0/1 (0)	0/3 (0)

**NOTE.** ZN, Ziehl-Neelsen staining; TB, tuberculosis; Gr. infl, granulomatous inflammation; Supp. infl, suppurative inflammation.

* Morphological features consistent with tuberculosis. 16% HIV positive.

** 31% HIV positive.

## Discussion

This is the first study to show that the immunochemistry-based MPT64 test, applied on human specimens from patients with presumptive EPTB, can be implemented in a low-resource routine diagnostic setting leading to significant improvement in the diagnosis of EPTB. The results are comparable with previous clinical studies performed in more controlled settings, especially for TB lymphadenitis [5, 25]. The overall performance of the MPT64 test was better compared to the other diagnostic tests, with a sensitivity of 83% in the confirmed TB cases.

In FNAC specimens from lymph nodes, the MPT64 test was positive in 76% of the TB cases (confirmed, probable and possible TB cases) compared to none of the non-TB cases, demonstrating high sensitivity and excellent specificity. The superior performance of the MPT64 test for diagnosing TB lymphadenitis using FNAC specimens can have important clinical implications. FNAC is a simple, safe, cost-effective, minimally invasive procedure ideal for use in resource-limited settings [26, 27]. The procedure can be performed in out-patient settings, also in peripheral areas. Fixed slides can then be transported to a hospital with diagnostic facilities for performing cytological evaluation [26, 27]. Further, the possibility of FNAC to distinguish TB and malignant disease is very important [28], as empirical use of ATT in patients with peripheral lymphadenopathy may lead to undue delay of a malignant diagnosis. In the current study, cytological evaluation of FNAC reported suspected malignancy in 10/66 (15%) patients presenting with peripheral lymphadenopathy.

The cytomorphological features in patients with TB lymphadenitis varied greatly in our study, and only 56% of TB cases had morphological features consistent with TB infection, even if most patients were HIV negative, implying the limited use of cytology for an accurate diagnosis of TB. This emphasises the need of additional tests. AFB microscopy does not distinguish between the *M*. *tuberculosis* and NTM, and has low sensitivity in TB lymphadenitis [25, 29]. Even though culture remains the gold standard of diagnosis, the need for advanced laboratory facilities and the long turnaround time is a challenge in resource-limited settings. The MPT64 test could provide a rapid and confirmative diagnosis of TB lymphadenitis using FNAC specimens, where culture results are absent or takes weeks to be completed. In the current study, all culture positive FNAC from lymph nodes were positive with the MPT64 test.

The sensitivity of the MPT64 test was significantly higher in children than in adults. This could be biased by the higher proportion of TB lymphadenitis cases amongst the children. Still, the sensitivity of the MPT64 test in FNAC specimens from lymph nodes was better in children than in adults (100% vs. 65%). FNAC has been suggested as the diagnostic modality of choice also in children [27]. In the recent years childhood TB has received increased attention, and global estimates imply that the diagnosis of TB is often missed in children and only one third of children developing active TB are notified [30]. In endemic areas, peripheral lymphadenitis is the most common extra-thoracic site of TB in children [31]. The MPT64 test could therefore be very useful in the correct diagnosis of TB lymphadenitis among children.

In the present study, we have also evaluated the MPT64 test according to HIV status, and found no significant difference in sensitivity or specificity when comparing HIV negative to HIV positive patients, implying that the MPT64 test could have an important clinical impact also in this patient group. However, because of the low number of HIV positive cases (n = 20) in this study, the test needs to be evaluated using a larger sample size.

Developing new laboratory diagnostic tests for EPTB is demanding, because of the range of various specimens, challenges with obtaining adequate samples, defining optimal sample volumes, the diverse ways of sample processing and the problem of imperfect reference standards. Culture is still used as the gold standard, but is known to be of limited value in EPTB [12, 13], which makes it difficult to evaluate a new diagnostic test. Using a suboptimal reference standard may potentially misclassify patients as TB or non-TB cases and bias the results of the test under evaluation [32]. To overcome this challenge, we chose to compare the MPT64 test with a CRS and the patients were categorized according to this CRS (Table 1). Culture and Xpert assay results were available in 86% and 50% of the specimens included in the data analysis. Only 8 specimens were positive with culture and 6 specimens were positive with the Xpert assay. The CRS classified 64 patients as TB cases. Therefore, using only culture as a reference standard would have underestimated the true value of the MPT64 test. The low sensitivity of culture in this study could partly be explained by loss of viable bacilli during transport to PHL-IdC at Pemba, the paucibacillary nature of EPTB disease and the possibility of uneven distribution of bacilli in the specimens sent to analyses. Further, two patients had started ATT for 5 and 17 days before specimens were collected, influencing the bacterial viability.

The evaluation of the Xpert assay is challenging in our study, as only 50% of the specimens were examined with this method. A previous study described a sensitivity of 70.6% in lymph nodes when the Xpert assay was compared against culture [33]. In a review, a pooled sensitivity of the Xpert assay in lymph node samples was reported to be 83.1% when compared against culture and 81.2% when using a CRS as a reference standard [12]. In the current study only 5 lymph node samples were culture positive, of these 3/4 (75%) were positive with the Xpert assay. Even though the numbers are low, one could get an impression that the sensitivity of the Xpert assay compared to culture is comparable to other studies using culture as a reference standard. The reason for the low sensitivity of the Xpert assay compared to the CRS in the current study could be due to different criteria incorporated in the CRS in our study and other studies reporting a higher sensitivity of the Xpert assay assessed against a CRS.

There are some limitations of this study. The sample size is small which makes it difficult to do further subgroup analysis of the performance of the MPT64 test according to all presumptive sites of infection. Secondly, there is a heterogeneity in the number of tests performed in patients with different types of EPTB clinical presentation. This is due to the study design, where the new MPT64 test was evaluated for its performance in the routine, without interfering with other routine diagnostic procedures. All samples were not subjected to all routine diagnostic methods due to various reasons. This may have influenced the performance of the component tests and the new test under assessment. Thirdly, the CRS may have reduced specificity, as defining a TB case based on clinical presumptive EPTB and response to ATT does not provide an accurate diagnosis of TB. It was therefore decided to subdivide the TB cases into “confirmed”, “probable” and “possible” TB cases and present the results of the various diagnostic tests for the separate groups.

## Conclusions

The MPT64 test is a robust, rapid, sensitive, and specific test for the etiological diagnosis of EPTB. It can differentiate between *Mycobacterium tuberculosis* complex species and NTM, and performs better than conventional methods and the Xpert assay. The test is particularly useful in correct diagnosis of TB lymphadenitis and in childhood TB, and performs equally well in HIV infected patients. Like any diagnostic test it should be interpreted together with the clinical history, examination and routine investigations. We show that the MPT64 test can be implemented in a routine laboratory in a low-resource setting, where improved diagnostics may have a valuable impact on patient management and outcome.

## Supporting information

S1 File Dataset. (SAV)

S1 Text Study questionnaire, English version (patients ≥ 18 years). (PDF)

S2 Text Study questionnaire, English version (patients < 18 years). (PDF)

S3 Text Study questionnaire, Swahili version (patients ≥ 18 years). (PDF)

S4 Text Study questionnaire, Swahili version (patients < 18 years). (PDF)
