# Supplementary material for: MPT64 antigen detection test improves routine diagnosis of extrapulmonary tuberculosis in a low-resource setting: A study from the tertiary care hospital in Zanzibar
Source: PLoS One. 2018 May 9;13(5):e0196723. doi: 10.1371/journal.pone.0196723 (PMC5942825; doi:10.1371/journal.pone.0196723)
Supplement: S1 Text — (PDF) [file pone.0196723.s002.pdf]

S1 Text. Study questionnaire, English version (patients ≥ 18 years).

Date:

Consultant:

Hospital:

Department: ☐ OPD ☐ IPD

☐ **Written informed consent** ☐ **Oral informed consent**

PATIENT IDENTIFICATION

Study Number: ________________

Age________Years

Gender: ☐ Male ☐ Female

Respondent: ☐ Patient ☐ Parent ☐ Spouse ☐ Child ☐ Other, relative/friend

Address: Region__________________ Village_______________

1. Marital status:

☐ Single ☐ Married ☐ Widow/widower ☐ Separated

☐ Divorced ☐ Cohabited ☐ Other, please specify____________

2. Level of education:

☐ No formal education ☐ Not completed primary school

☐ Completed primary school ☐Form I-IV

☐ Form IV-VI ☐Above secondary education

☐Adult education ☐Others (Please mention)______

3. Religion:

☐Muslim ☐ Christian ☐ Other, please mention____________

PREVIOUS MEDICAL HISTORY

4. Tobacco use: ☐ Yes ☐ No ____weeks/months /years

5. Smoking: ☐ Yes ☐ No ____weeks/months /years

6. Alcohol: ☐ Yes ☐ No ____weeks/months /years

7. Associated Diseases

COPD: ☐ Yes ☐ No

Renal Disease: ☐ Yes ☐ No

Liver Diseases: ☐ Yes ☐ No

Diabetes Mellitus: ☐ Yes ☐ No

Hypertension: ☐ Yes ☐ No

Other: ☐ Yes ☐ No

Describe other: _____________________________

____

8. Medication: ***Please write the names of the medication***

____

PREVIOUS HISTORY OF TUBERCULOSIS

9. Have you been in contact with a person with known tuberculosis?

☐ Yes ☐ No

10. Have you previously been diagnosed with pulmonary tuberculosis?

☐ Yes ☐ No

11. Have you previously been diagnosed with extrapulmonary tuberculosis:

☐ Yes ☐ No

If yes, please specify: ____

12. Have you previously been treated for tuberculosis?

☐ Yes ☐ No

13. If you have been treated, what was the treatment outcome?

☐ Cured

☐ Treatment Completed

☐ Treatment Interrupted

14. If treated, when was the last time that you completed any TB treatment? _________________

HEALTH-SEEKING BEHAVIOUR AND DIAGNOSTIC DELAY

Health seeking behavior for TB patients
Please remind the patient that this survey is confidential.

15. Please ask if the patient has experienced any of the following symptoms

General Symptoms

Fever: ☐ Yes ☐ No ____weeks/months
What kind of fever do you have?
☐ High-grade ☐ Low-grade
When do you have fever?
☐ Morning ☐ Day-time ☐ Evening ☐ Night ☐ all day
Loss of weight: ☐ Yes ☐ No ____weeks/months
Loss of appetite: ☐ Yes ☐ No ____weeks/months
Night Sweat: ☐ Yes ☐ No ____weeks/months
Fatigue: ☐ Yes ☐ No ____weeks/months
Amenorrhea/female: ☐ Yes ☐ No ____weeks/months
Body weakness: ☐ Yes ☐ No ____weeks/months
Frequent cold: ☐ Yes ☐ No ____weeks/months
Neck mass: ☐ Yes ☐ No ____weeks/months
Other: ☐ Yes ☐ No ____weeks/months

Respiratory Symptoms

Cough: ☐ Yes ☐ No ___weeks/months
Sputum: ☐ Yes ☐ No ___weeks/months
Cough with Sputum: ☐ Yes ☐ No ___weeks/months
Cough with blood: ☐ Yes ☐ No ___weeks/months
Chest pain: ☐ Yes ☐ No ___weeks/months
Difficult in breathing: ☐ Yes ☐ No ___weeks/months

Abdominal Symptoms

Swelling of/in stomach: ☐ Yes ☐ No ___weeks/months
Fullness of the stomach: ☐ Yes ☐ No ___weeks/months
Vomiting: ☐ Yes ☐ No ___weeks/months
Diarrhea: ☐ Yes ☐ No ___weeks/months
Other: ☐ Yes ☐ No ___weeks/months
Describe other: ____

____

\

Neurological Symptoms

Headache: ☐ Yes ☐ No ___weeks/months
Photophobia: ☐ Yes ☐ No ___weeks/months
Vomiting: ☐ Yes ☐ No ___weeks/months
Dizziness: ☐ Yes ☐ No ___weeks/months
Vertigo: ☐ Yes ☐ No ___weeks/months
Weakness/Numbness of extremity:
☐ Yes ☐ No ___weeks/months
Visual disturbance: ☐ Yes ☐ No ___weeks/months

Other: □ Yes □ No ___weeks/months
Describe other: ______________________________
____
____

16. What were the major symptoms that first made you seek care?
□ Prolong Cough □ coughing blood □ Breathlessness
□ Chest pain □ Fever □ Weight loss
□ Fatigue\Weakness □ Loss of appetite □ Night sweats
□ Bone pain □ Lymph node swelling □ Diarrhoea
□ abdominal pain □ others (specify) __________________

17. When did you first notice the symptoms?
____

18. Did you practice any self-medication before you sought care?
□ Yes □ No

19. When did you first seek medical advice for this illness after noticing the symptoms? (Ask the respondent to specify how many days/weeks after noticing the symptoms)
□ Today □ 1-6 days □ 1-4 weeks □ 5-8weeks □ over 8 weeks

20. How many different places did you go to seek help for the current symptoms? (Ask the respondent to specify the various health care providers/health facilities)

________________places

21. How many times have you visited health facilities with the same symptoms before?
□ First visit □ Second visit □ Third visit
□ > 3 visits □ don't remember

22. Which place did you first seek care for your symptoms?
□ Regional Hospital □ District hospital/PHCC □ Health center/PHCU
□ Dispensary/private clinic □ Private Hospital □ Traditional healer
□ Pharmacy □ other, please specify______________________________

23. Did you get any medicine from there?
□ Yes □ No

24. If yes, what kind of medicine?
□ Antibiotics □ Anti-TB □ Herbs □ others, which______________

25. Were the symptoms relieved after taking medicines?
□ Yes □ No

26. What kind of diagnosis did you receive for your illness? _________________________

27. Were any tests done at the first medical service?
□ Yes □ No

28. What type of tests?
□ Blood test □ Urine test □ Sputum □ X-ray
□Others, please specify_________________________________

29. Did you take the results back to the doctor?
☐ Yes ☐ No

30. Could you estimate the total cost for the previous visits/investigations related to your current illness?
Admission ____________________________TZS
Consultations ____________________________ TZS
Medication ____________________________TZS
Laboratory tests/X-ray/CT___________________________TZS
Transportation ____________________________TZS

31. Who referred you here to this health facility?
☐ myself ☐ Traditional healers ☐ Religious leaders
☐ Pharmacy/drug shop ☐ Village health worker ☐ Government dispensary
☐ Government health center ☐ Government hospital ☐ Private dispensary/hospital
☐ Charitable/NGO ☐ Member of the family ☐ Other_____________________

32. Have you ever been tested for HIV?
☐ Yes ☐ No

33. Self-reported HIV results
☐ HIV positive ☐ HIV negative ☐ don't know ☐ don't agree to disclose HIV status

34. Before today, had you heard of the illness tuberculosis?
☐ Yes ☐ No

35. Do you know any symptoms of tuberculosis?
☐ Chronic cough ☐ Spitting blood ☐ Shortness of breath
☐ Chest pain ☐ Fever ☐Weight loss
☐ Tiredness ☐ Loss of appetite
☐ Others Please specify________________________________ ***(Do not probe but ask for more symptoms)***

36. Do you know which parts of the body that can be affected by tuberculosis?
____

37. Can tuberculosis spread from person to person?
☐ Yes ☐ No ☐ Uncertain

38. Do you drink unboiled milk?
☐ Yes ☐ No

39. Do you eat raw meat?
☐ Yes ☐ No

40. Did you know that consumption of raw animal products, like uncooked dairy products can lead to gastrointestinal tuberculosis as a result of transfer of the disease from animals to humans?
☐ Yes ☐ No

41. Can tuberculosis be cured with medicines?
☐ Yes ☐ No ☐ Uncertain

42. Do you know how long it takes to treat tuberculosis?
☐ Yes ☐ No
If yes, do you know the approximate duration of treatment? ______________________

43. Do people in your community associate tuberculosis with HIV?
☐ Yes ☐ No ☐ Uncertain
If yes, why do they associate it with HIV? ___________________________________

44. Is there anything that would make it easier for people with tuberculosis to get treatment, not just in this clinic, but in other health facilities?
☐ Yes ☐ No ☐ Uncertain
If yes, what could be done? ____

45. The moment you realized you may have contracted tuberculosis, did you have any problems deciding to seek care? If so, what types of problems?
____
____
____

46. What fears do others have about TB that prevents them from seeking medical advice?
____

47. If you consulted a traditional healer before seeking care at a modern health facility, what were the reasons which led you to first use the traditional healer?

PATIENT AND HOUSEHOLD COSTS, estimate of the patient income level

48. How long does it take you to go to the nearest health facility?
□ Less than 30 minutes □ between 30 minutes and one hour □ More than one hour

49. How far is this hospital to your home (in Kilometers) _________________

50. How long (on average) does it take you to this health facility, waiting for your consultation and finally returning to your home\workplace? ________________ Hours

51. How did you get to this health facility?
□ Walked □ Bicycle □Motorcycle □ Private car □ Dala Dala

52. If you have to take a Dala Dala, how much (on average) does it cost you to come to the clinic? ________________TZS.

53. Do you usually have to make some special arrangements at home before coming to the clinic?
□ Yes □ No □ Uncertain
If yes, what arrangements?__________________________

54. What is your main occupation (past twelve months)?
□ Employed by government
□ Employed private for profit sector
□ Employed by NGO
□ Self-employed, business with employees
□ Self-employed, business no employee
□ Self-employed (merchant), farmer/ fishing
□ Unemployed
□ Retired
□ Pupil/ student
□ Disabled/ sick
□ Housewife
□ Other_________________

55. What is the main income of your house hold?
□ Crop production
□ Livestock
□ Fishing
□ Hunting/ bee-keeping
□ Poultry
□ Farm wage
□ Other agricultural activity
□ Wages (government)
□ Wages (private)
□ Monetary savings (interest)
□ Pensions
□ Property (rentals)
□Self-employed payments (merchant)
□Other Specify____________________________

56. In the past 12 months, in what types of activities were you and any members of your household engaged? (Only income-generating activities)?
____
____
____

57. How much did (NAME) earn (money) for the activities stated on average in the past 12 months? This should include not only salary or cash income: but also the value of goods produced or traded for other goods and services.
____

58. Do you have reduced working capacity due to your current illness?
☐ Yes, completely stopped working ☐ Yes, working but with reduced capacity
☐ Working as normal

59. If you have reduced working capacity, how would you describe your reduced working capacity?
____

60. When did you stop working or working with reduced capacity?
____

61. Have any member of your household stopped working or reduced their work capacity because of your illness?
☐ Yes ☐ No
If yes, for how long? _______________days
If yes, how much reduced working capacity? ____________________________________

62. Have you/or any member of your household lost any wages or income because of your illness?
☐ Yes ☐ No ☐ Uncertain
If yes, how much_____________________

63. Do you own a house?
☐ Yes ☐ renting a house ☐ living with relatives /friends ☐ Homeless

64. How many people live in your household: __________________ (number of people)
How many: Men: _______Women:_______Elderly:_______Children (between 0-10):_______
Children (between 11-18):_______

65. What is the main source of drinking water for members of your household?
☐ Piped water 1=Piped into dwelling 2= Piped into yard/plot 3=Public tap 4=Neighbors' tap
☐ Water from open well
☐ Water from covered well or borehole
☐ Running water 1=spring; 2=river/stream; 3=pond/Lake; 4=Dam
☐ Rain water
☐ Tanker truck
☐ Water vendor
☐ Bottled water
☐ Others Specify _________________________

66. What kind of toilet facilities does your household have?
☐ Flush toilet ☐ Pit toilet/latrine 1=traditional pit latrine 2=ventilated pit latrine (VIP)
☐ No facility/bush/field ☐ other, please specify _______________

67. Do you share these facilities with other households?
☐ Yes ☐ No

68. Does your household have?
☐ Electricity ☐ Paraffin lamp ☐ Radio
☐ Television ☐ Telephone/mobile ☐ Iron (either charcoal or electricity)
☐ Refrigerator

69. What is the main source of energy for lighting in your household?
☐ Main electricity ☐ Solar ☐ Gas
☐ Paraffin-hurricane lamp ☐ Paraffin-Wick lamp ☐ Firewood
☐ Candles ☐ other, please specify __________________________

70. What is the main material for the walls of your house or house you are living?

☐ Grass ☐ Poles and mud ☐ Cement bricks
☐ Backed bricks ☐ Timber ☐ Stones
☐ Others Specify___________________________

71. What is the roofing material of your house or house you are living?

☐ Grass/leaves/mud ☐ Iron sheets ☐ Tiles ☐ Concrete ☐ Asbestos
☐ Others Specify________________

72. Does any member of your household own

☐ A bicycle ☐ A motorcycle or motor scooter ☐ A car ☐ A bank account

73. How many acres of land for farming/grazing are owned by the household?

☐ Arable land______________acres ☐ Land for grazing___________acres

74. How many meals does your household usually have per day?

Meals__________________
