# Supplementary material for: MPT64 antigen detection test improves routine diagnosis of extrapulmonary tuberculosis in a low-resource setting: A study from the tertiary care hospital in Zanzibar
Source: PLoS One. 2018 May 9;13(5):e0196723. doi: 10.1371/journal.pone.0196723 (PMC5942825; doi:10.1371/journal.pone.0196723)
Supplement: S2 Text — (PDF) [file pone.0196723.s003.pdf]

S2 Text. Study questionnaire, English version (patients < 18 years).

Date:

Consultant:

Hospital:

Department: ☐ OPD ☐ IPD

☐ **Written informed consent** ☐ **Oral informed consent**

PATIENT IDENTIFICATION

Study Number: __________________

Age________Years

Gender: ☐ Male ☐ Female

Respondent: ☐ Patient ☐ Parent ☐ Other, please specify_______________________

Address: Region___________________ Village________________

1. Living with:

☐ one parent (mother/father) ☐ both parents ☐ Relatives ☐ Orphanage

☐ Others, please specify_____________________

2. Education/day-care:

☐ home-care ☐ nursery ☐ primary school

☐ Form I-IV ☐ Form IV-VI ☐Others (Please mention) ______

3. Religion:

☐Muslim ☐ Christian ☐ other, please mention_____________

PREVIOUS MEDICAL HISTORY

4. Tobacco use (adolescents): ☐ Yes ☐ No ____weeks/months /years

5. Smoking (adolescents): ☐ Yes ☐ No ____weeks/months /years

6. Alcohol (adolescents): ☐ Yes ☐ No ____weeks/months /years

7. Associated Diseases

Asthma: ☐ Yes ☐ No

Chronic diarrhoea ☐ Yes ☐ No

Renal Disease: ☐ Yes ☐ No

Liver Diseases: ☐ Yes ☐ No

Diabetes Mellitus: ☐ Yes ☐ No

Cardiac disease: ☐ Yes ☐ No

Other: ☐ Yes ☐ No

Describe other: _______________________________

____

8. Medication: ***Please write the names of the medication***

____

PREVOIUS HISTORY OF TUBERCULOSIS

9. Has the child been in contact with a person with known tuberculosis?

 Yes No

If yes, who was/were the contact person(s)?

____

If yes, when was the child in contact with this (these) person(s)?

____

10. Has the child previously been diagnosed with pulmonary tuberculosis?

 Yes No

11. Has the child previously been diagnosed with extrapulmonary tuberculosis?

 Yes No

12. Has the child previously been treated for tuberculosis?

 Yes No

13. If the child has been treated, what was the treatment outcome?

 Cured
 Treatment Completed
 Treatment Interrupted

14. If treated, when was the last time the child completed any TB treatment? ___________________

HEALTH-SEEKING BEHAVIOUR AND DIAGNOSTIC DELAY

Health seeking behavior for TB patients
Please remind the patient and respondent that this survey is confidential.

15. Please ask if the patient has experienced any of the following symptoms

General Symptoms

Fever: □ Yes □ No ____weeks/months

What kind of fever does the child have?
□ High-grade □ Low-grade

When does the child have fever?
□ Morning □ Day-time □ Evening □ Night □ all day

Failure to gain weight □ Yes □ No ____weeks/months
Loss of weight: □ Yes □ No ____weeks/months
Loss of appetite: □ Yes □ No ____weeks/months
Night Sweat: □ Yes □ No ____weeks/months
Fatigue: □ Yes □ No ____weeks/months
Body weakness: □ Yes □ No ____weeks/months
Frequent cold: □ Yes □ No ____weeks/months
Neck mass: □ Yes □ No ____weeks/months
Other: □ Yes □ No ____weeks/months

Respiratory Symptoms

Cough: □ Yes □ No ___weeks/months
Sputum: □ Yes □ No ___weeks/months
Cough with Sputum: □ Yes □ No ___weeks/months
Cough with blood: □ Yes □ No ___weeks/months
Chest pain: □ Yes □ No ___weeks/months
Difficult in breathing: □ Yes □ No ___weeks/months

Abdominal Symptoms

Swelling of/in stomach: □ Yes □ No ___weeks/months
Fullness of the stomach: □ Yes □ No ___weeks/months
Vomiting: □ Yes □ No ___weeks/months
Chronic diarrhea: □ Yes □ No ___weeks/months
Other: □ Yes □ No ___weeks/months
Describe other: ____

____

\

Neurological Symptoms

Headache: □ Yes □ No ___weeks/months
Irritability □ Yes □ No ___weeks/months
Photophobia: □ Yes □ No ___weeks/months
Vomiting: □ Yes □ No ___weeks/months
Dizziness: □ Yes □ No ___weeks/months
Vertigo: □ Yes □ No ___weeks/months
Weakness/Numbness of extremity:
□ Yes □ No ___weeks/months

Visual disturbance: ☐ Yes ☐ No ___weeks/months
Other: ☐ Yes ☐ No ___weeks/months
Describe other: ______________________________
____
____

16. What were the major symptoms that first made you seek care for your child?
☐ Prolong Cough ☐ Coughing blood ☐ Breathlessness
☐ Chest pain ☐ Fever ☐ Weight loss
☐ Fatigue\Weakness ☐ Loss of appetite ☐ Night sweats
☐ Bone pain ☐ Lymph node swelling ☐ Diarrhoea
☐ Abdominal pain ☐ Others (specify) _________________

17. When did you or the child first notice the symptoms?
____

18. Did you give the child any self-medication before you sought care?
☐ Yes ☐ No

19. When did you first seek medical advice for the child after noticing the symptoms? (Ask the respondent to specify how many days/weeks after noticing the symptoms)
☐ Today ☐ 1-6 days ☐ 1-4 weeks ☐ 5-8weeks ☐ over 8 weeks

20. How many different places did you go to seek help for the child's current symptoms? (Ask the respondent to specify the various health care providers/health facilities)

______________places

21. How many times have you taken the child to a health facility with the same symptoms before?
☐ First visit ☐ Second visit ☐ Third visit
☐ > 3 visits ☐ don't remember

22. Which place did you first seek care for the child`s symptoms?
☐ Regional Hospital ☐ District hospital/PHCC ☐ Health center/PHCU
☐ Dispensary/private clinic ☐ Private Hospital ☐ Traditional healer
☐ Pharmacy ☐ other, please specify_____________________________

23. Did the child get any medicine from there?
☐ Yes ☐ No

24. If yes, what kind of medicine?
☐ Antibiotics ☐ Anti-TB ☐ Herbs ☐ others, which_____________

25. Were the child`s symptoms relieved after taking medicines?
☐ Yes ☐ No

26. What kind of diagnosis did the child receive for the current illness? ________________________

27. Were any tests done at the first medical service?
☐ Yes ☐ No

28. What type of tests?
☐ Blood test ☐ Urine test ☐ Sputum ☐ X-ray

□Others, please specify________________________________

29. Did you take the results back to the doctor?
□ Yes □ No

30. Could you estimate the total cost for the previous visits/investigations related to the child`s current illness?
Admission __________________________TZS
Consultations __________________________ TZS
Medication __________________________TZS
Laboratory tests/X-ray/CT_________________________TZS
Transportation __________________________TZS

31. Who referred the child here to this health facility?
□ myself □ Traditional healers □ Religious leaders
□ Pharmacy/drug shop □ Village health worker □ Government dispensary
□ Government health center □ Government hospital □ Private dispensary/hospital
□ Charitable/NGO □ Member of the family □ Other____________________

32. Has your child received routine vaccination, by following the national children`s vaccination program?
□ Yes □ No

33. Has your child received BCG vaccination?
□ Yes □ No

34. Has the child ever been tested for HIV?
□ Yes □ No

35. What was the result of the HIV-test?
□ HIV positive □ HIV negative □ don't know □ don't agree to disclose HIV status

36. Before today, had you heard of the illness tuberculosis?
□ Yes □ No

37. Do you know any symptoms of tuberculosis?
□ Chronic cough □ Spitting blood □ Shortness of breath
□ Chest pain □ Fever □Weight loss
□ Tiredness □ Loss of appetite
□ Others Please specify______________________________ ***(Do not probe but ask for more symptoms)***

38. Do you know which parts of the body that can be affected by tuberculosis?
____

39. Can tuberculosis spread from person to person?
□ Yes □ No □ Uncertain

40. In your household do you drink unboiled milk?
□ Yes □ No

41. In your household do you eat raw meat?
□ Yes □ No

42. Did you know that consumption of raw animal products, like uncooked dairy products can lead to gastrointestinal tuberculosis as a result of transfer of the disease from animals to humans?
☐ Yes ☐ No

43. Can tuberculosis be cured with medicines?
☐ Yes ☐ No ☐ Uncertain

44. Do you know how long it takes to treat tuberculosis?
☐ Yes ☐ No
If yes, do you know the approximate duration of treatment? ______________________

45. Do people in your community associate tuberculosis with HIV?
☐ Yes ☐ No ☐ Uncertain
If yes, why do they associate it with HIV? **__________________________________**

46. Is there anything that would make it easier for people with tuberculosis to get treatment, not just in this clinic, but in other health facilities?
☐ Yes ☐ No ☐ Uncertain
If yes, what could be done? ____

47. The moment you realized that the child may have contracted tuberculosis, did you have any problems deciding to seek care? If so, what types of problems?
____
____
____

48. What fears do others have about TB that prevents them from seeking medical advice?
____

49. If you consulted a traditional healer before seeking care at a modern health facility, what were the reasons which led you to first use the traditional healer?

PATIENT AND HOUSEHOLD COSTS, estimate of the patient income level

50. How long does it take you to go to the nearest health facility?
 Less than 30 minutes between 30 minutes and one hour More than one hour

51. How far is this hospital to your home (in Kilometers) _________________

52. How long (on average) does it take you to this health facility, waiting for the child`s consultation and finally returning to your home\workplace? ________________ Hours

53. How did you get to this health facility?
 Walked Bicycle Motorcycle Private car Dala Dala

54. If you have to take a Dala Dala, how much (on average) does it cost you to come to the clinic? ________________TZS.

55. Do you usually have to make some special arrangements at home before coming to the clinic?
 Yes No Uncertain
If yes, what arrangements? **__________________________**

56 What is the main income of your household?

Crop production	Livestock	Fishing
Hunting/ bee-keeping	Poultry	Farm wage
Other agricultural activity	Wages (government)	Wages (private)
Monetary savings (interest)	Pensions	
Property (rentals)	Self-employed payments (merchant)	

Other Specify____________________________

57. In the past 12 months, in what types of activities were you and any members of your household engaged? (Only income-generating activities)?
____
____
____

58. How much did (NAME) earn (money) for the activities stated on average in the past 12 months? This should include not only salary or cash income: but also the value of goods produced or traded for other goods and services.
____

59. Have any member of your household stopped working or reduced their work capacity because of the child`s illness?
 Yes No
If yes, for how long? _______________days
If yes, how much reduced working capacity? __________________________________

60. Have you/or any member of your household lost any wages or income because of the child`s illness?
 Yes No Uncertain
If yes, how much____________________

61. Do you own a house?

□ Yes □ Renting a house □ living with relatives /friends □ Homeless

62. How many people live in your household: ________________ (number of people)
How many: Men: _______Women:_______Elderly:_______Children (between 0-10):_______
Children (between 11-18): _______

63. How many siblings does the child have?
__________sibling(s)

64. What is the main source of drinking water for members of your household?
□ Piped water 1=Piped into dwelling 2= Piped into yard/plot 3=Public tap 4=Neighbors' tap
□ Water from open well
□ Water from covered well or borehole
□ Running water 1=spring; 2=river/stream; 3=pond/Lake; 4=Dam
□ Rain water
□ Tanker truck
□ Water vendor
□ Bottled water
□ Others Specify ________________________

65. What kind of toilet facilities does your household have?
□ Flush toilet □ Pit toilet/latrine 1=traditional pit latrine 2=ventilated pit latrine (VIP)
□ No facility/bush/field □ other, please specify ______________

66. Do you share these facilities with other households?
□ Yes □ No

67. Does your household have?
□ Electricity □ Paraffin lamp □ Radio
□ Television □ Telephone/mobile □ Iron (either charcoal or electricity)
□ Refrigerator

68. What is the main source of energy for lighting in your household?
□ Main electricity □ Solar □ Gas
□ Paraffin-hurricane lamp □ Paraffin-Wick lamp □ Firewood
□ Candles □ other, please specify _________________________

69. What is the main material for the walls of your house or house you are living?
□ Grass □ Poles and mud □ Cement bricks
□ Backed bricks □ Timber □Stones
□ Others Specify_________________________

70. What is the roofing material of your house or house you are living?
□ Grass/leaves/mud □ Iron sheets □ Tiles □ Concrete □ Asbestos
□ Others Specify_______________

71. Does any member of your household own
□ A bicycle □ A motorcycle or motor scooter □ A car □ A bank account

72. How many acres of land for farming/grazing are owned by the household?
□ Arable land_____________acres □ Land for grazing__________acres

73. How many meals does your household usually have per day?
Meals_________________
