# Supplementary material for: MPT64 antigen detection test improves routine diagnosis of extrapulmonary tuberculosis in a low-resource setting: A study from the tertiary care hospital in Zanzibar
Source: PLoS One. 2018 May 9;13(5):e0196723. doi: 10.1371/journal.pone.0196723 (PMC5942825; doi:10.1371/journal.pone.0196723)
Supplement: S3 Text — (PDF) [file pone.0196723.s004.pdf]

S3 Text. Study questionnaire, Swahili version (patients ≥ 18 years).

DODOSO – Vijana wenye umri zaidi ya miaka 18 na watu wazima

Tarehe:

Mshauri Bingwa:

Hospitali:
Idara: Idara ya wagonjwa wa Nje Idara ya wagonjwa wa Ndani

 Taarifa ridhaa ya maandishi **Taarifa ridhaa ya mdomo**

UTAMBULISHO WA MGONJWA:
Namba ya uchunguzi _________________
Umri miaka ________
Jinsia: ME KE
Mhusika: Mgonjwa Mzazi Mke/ Mume Mtoto
 Wengineo, tafadhali taja ______________________
Anwani: Mkoa __________________ Kijiji _______________

1. Hali ya Ndoa:
 Sijaoa/sijaolewa Nimeoa/nimeolewa Mgane/mjane Tumeachana
 Tumetengana Kinyumbana Hali nyingine, tafadhali taja ____________

2. Kiwango cha Elimu:
 Hakuna elimu ya darasani Sikumaliza elimu ya msingi
 Nilimaliza elimu ya msingi Kati ya kidato cha I – IV
 Kidato cha IV – VI Zaidi ya elimu ya sekondari
Elimu ya watu wazima Nyinginezo, tafadhali taja _____________

3. Dini Madhehebu
 Muislam Mkristo Nyingineyo tafadhali taja ____________________

HISTORIA YA MATIBABU BINAFSI NA SIKU ZA NYUMA
4. Matumizi ya tumbaku Ndio Hapana ____wiki/miezi/miaka
5. Uvutaji Ndio Hapana ____wiki/miezi/miaka
6. Utumiaji wa pombe Ndio Hapana ____wiki/miezi/miaka

7. Magonjwa husishwa
Ugonjwa sugu wa mapafu: Ndio Hapana
Ugonjwa wa figo: Ndio Hapana
Ugonjwa wa Ini: Ndio Hapana
Kisukari: Ndio Hapana
Shinikizo la damu: Ndio Hapana
Mengineyo: Ndio Hapana
Eleza mengineyo: ______________________________
____

8. Dawa: *Tafadhali andika aina ya dawa ulizo/unazo tumia*

____

HISTORIA YA UGONJWA WA KIFUA KIKUU

9. Je, umewahi kukaribiana au kuwa na mtu anayejulikana kuwa na Kifua Kikuu?
 Ndio Hapana

10. Je, umewahi kuugua kifua kikuu?
 Ndio Hapana

11. Je, umewahi kuugua cha kifua kikuu nje ya mapafu?
 Ndio Hapana
Kama ndio, tafadhali toa maelezo: ____

12. Je, umewahi kutibiwa kifua kikuu?
 Ndio Hapana

13. Kama umewahi kutibiwa kifua kikuu, nini kilikuwa matokeo ya Matibabu?
 Nilipona
 Matibabu yalikamilika
 Matibabu yalikatizwa

14. Kama ulitibiwa, Ni lini mara ya mwisho ulikamilisha matibabu yoyote ya kifua kikuu?
____

TABIA YA UTAFUTAJI WA MATIBABU NA UCHELEWESHAJI WA UCHUNGUZI
Tabia ya utafutaji matibabu kwa wagonjwa wa kifua kikuu
Tafadhali mfahamishe/ mkumbushe mgonjwa/ mhusika kwamba utafiti huu ni wa siri

15. Tafadhali muulize mgonjwa kama ana uzoefu wowote wa dalili zifuatazo

Dalili za ujumla
Homa: ☐ Ndio ☐ Hapana ____wiki/miezi
Una aina gani ya Homa?
☐ Kiwango cha Juu ☐ Kiwango cha Chini
Ni wakati gani unakuwa na homa?
☐ Asubuhi ☐ Mchana ☐ Jioni ☐ Usiku ☐ Siku nzima
Kupungua uzito: ☐ Ndio ☐ Hapana ____wiki/miezi
Ukosefu wa Hamu ya Chakula: ☐ Ndio ☐ Hapana ____wiki/miezi
Utoaji Jasho Usiku: ☐ Ndio ☐ Hapana ____wiki/miezi
Uchovu: ☐ Ndio ☐ Hapana ____wiki/miezi
Kukosa siku za hedhi/wanawake ☐ Ndio ☐ Hapana ____wiki/miezi
Udhaifu wa Mwili ☐ Ndio ☐ Hapana ____wiki/miezi
Mafua ya mara kwa mara ☐ Ndio ☐ Hapana ____wiki/miezi
Uvimbe shingoni: ☐ Ndio ☐ Hapana ____wiki/miezi
Mengineyo: ☐ Ndio ☐ Hapana ____wiki/miezi

Dalili kwenye ya njia ya hewa
Kukohoa: ☐ Ndio ☐ Hapana ____wiki/miezi
Makohozi: ☐ Ndio ☐ Hapana ____wiki/miezi
Kikohozi chenye makohozi: ☐ Ndio ☐ Hapana ____wiki/miezi
Kukohoa damu: ☐ Ndio ☐ Hapana ____wiki/miezi
Maumivu ya kifua: ☐ Ndio ☐ Hapana ____wiki/miezi
Kushindwa kupumua: ☐ Ndio ☐ Hapana ____wiki/miezi

Dalili kwenye ya njia ya chakula
Kuvimba/Uvimbe tumboni: ☐ Ndio ☐ Hapana ____wiki/miezi
Tumbo kujaa: ☐ Ndio ☐ Hapana ____wiki/miez
Kutapika: ☐ Ndio ☐ Hapana ____wiki/miezi
Kuharisha: ☐ Ndio ☐ Hapana ____wiki/miezi
Mengineyo: ☐ Ndio ☐ Hapana ____wiki/miezi
Elezea dalili nyinginezo: ____

____

\

Dalili kwenye mfumo wa fahamu
Maumivu ya Kichwa: ☐ Ndio ☐ Hapana ____wiki/miezi
Macho kuogopa mwanga: ☐ Ndio ☐ Hapana ____wiki/miezi
Kutapika: ☐ Ndio ☐ Hapana ____wiki/miezi
Kizunguzungu: ☐ Ndio ☐ Hapana ____wiki/miezi
Maruweruwe: ☐ Ndio ☐ Hapana ____wiki/miezi
Udhaifu wa mwili/miguu na mikono kufa ganzi:
☐ Ndio ☐ Hapana ____wiki/miezi
Matatizo ya macho: ☐ Ndio ☐ Hapana ____wiki/miezi

Mengineyo: ☐ Ndio ☐ Hapana ____wiki/miezi

Eleza dalili nyinginezo: ______________________________
____
____

16. Ni dalili zipi za ugonjwa ambazo zilikufanya utafute matibabu ya mwanzo?

☐ Kikohozi cha muda mrefu ☐ Kukohoa damu ☐ Kushindwa kupumua
☐ Maumivu ya kifua ☐ Homa ☐ Kupungua uzito
☐ Udhaifu wa mwili/ Uchovu ☐ Kukosa hamu ya chakula ☐ Kutokwa jasho usiku
☐ Maumivu ya mifupa ☐ Kuvimba tezi ☐ Kuharisha
☐ Maumivu ya tumbo ☐ Mengineyo (Taja) dalili nyingine _________________

17. Lini uliziona dalili kwa mara ya kwanza?

____

18. Ulijipatia matibabu yoyote binafsi kabla ya kutafuta huduma ya matibabu ya Afya?

☐ Ndio ☐ Hapana

19. Lini kwa mara ya kwanza ulitafuta ushauri wa kimatibabu baada ya kugundua dalili?

☐ Leo ☐ Kati ya siku 1-6 ☐ kati ya wiki 1 – 4 ☐ kati ya wiki 5-8 ☐ zaidi ya wiki 8

20. Sehemu ngapi tofauti ulikwenda kutafuta msaada wa ajili ya dalili za sasa za ugonjwa?
Sehemu________________

21. Ni mara ngapi umekwenda kwenye huduma za afya ukiwa na dalili hizi za ugonjwa?

☐ Mara ya kwanza ☐ Mara mbili ☐ Mara ya tatu
☐ Zaidi ya mara tatu ☐ Sikumbuki

22. Sehemu ipi ya kwanza ulitafuta huduma ya afya kutokana na dalili zako za ugonjwa?

☐ Hospitali ya Mkoa ☐ Hospitali ya Wilaya/PHCC ☐ Kituo cha Afya/PHCU
☐ Zahanati ☐ Hospitali ya binafsi ☐ Mganga wa Jadi
☐ Duka la Dawa ☐ Nyinginezo, tafadhali taja _______________________________

23. Je, Ulipata dawa yoyote kutoka hapo?

☐ Ndio ☐ Hapana

24. Kama ndiyo, ulipata dawa aina gani?

☐ Antibakteria ☐ Dawa za kifua kikuu
☐ Miti Shamba ☐ Nyinginezo taja ____

25. Je dalili za ugonjwa zilipungua baada ya kutumia dawa?

☐ Ndio ☐ Hapana

26. Uligundulika una ugonjwa gani? ________________________

27. Kulikuwa na uchunguzi wowote wa kitabibu uliofanyika katika kituo cha kwanza cha afya ulichotembelea?

☐ Ndio ☐ Hapana

28. Ni aina gani ya vipimo?

☐ Kipimo cha damu ☐ Kipimo cha mkojo ☐ kipimo cha kikohozi ☐ Kipimo vya mionzi
☐ Vipimo vinginevyo, eleza ________________________________

29. Je uliyarudisha majibu ya vipimo kwa daktari?
□ Ndio □ Hapana

30. Je unaweza kukadiria gharama ulizotumia awali kwa kwenda vituo vya afya au kufanyiwa uchunguzi kuhusiana na ugonjwa wako wa sasa?

Usajili kumwona daktari ________________TZS
Kulazwa _______________ TZS
Dawa ________________TZS
Vipimo vya maabara/x-ray/CT _________________TZS
Usafiri _________________TZS

31. Nani aliyekupa rufaa kuja kwenye kituo hiki cha huduma za afya?
□ Mimi mwenyewe □ Mganga wa Jadi □ Viongozi wa kidini
□ Wauza madawa □ Wahudumu wa Afya wa kijiji □ Kituo cha Afya cha Serikali
□ Zahanati ya serikali □ Hospitali ya serikali □ Zahanati Binafsi / Hospitali
□ Vituo vya afya vya wahisani/mashirika yasiyo ya kiserikali □ Mwanafamilia
□ Wengineo: ___________________

32. Umewahi kufanyiwa kipimo cha VVU/UKIMWI?
□ Ndio □ Hapana

33. Taarifa binafsi za matokeo ya kipimo cha VVU/UKIMWI
□ Maambukizi □ Hakuna maambukizi □ Sijui □ Siwezi kusema hali yangu maambukizi

34. Kabla ya leo, ulikuwa umewahi kusikia ugonjwa wa kifua kikuu?
□ Ndio □ Hapana

35. Unazijua dalili zozote za ugonjwa wa kifua kikuu?
□ Kikohozi Sugu □ Kutema damu □ Kushindwa kupumua
□ Maumivu ya kifua □ Homa □ Kupungua uzito
□ Kuchoka □ Kukosa hamu ya chakula
□ Nyinginezo tafadhali taja _____________________________ ***(Usihoji bali uliza dalili zaidi za ugongwa wa kifua kikuu)***

36.Unajua ni sehemu gani mwilini zinazoweza kuathiriwa na kifua kikuu?
____

37. Je, ugonjwa wa kifua kikuu unaweza kuambukiza kutoka kwa mtu mmoja kwenda kwa mwingine?
□ Ndio □ Hapana □ Sina uhakika

38. Je, unakunywa maziwa yasiyochemshwa?
□ Ndio □ Hapana

39. Je, unakula nyama mbichi?
□ Ndio □ Hapana

40. Je ulijua kwamba utumiaji / ulaji wa bidhaa za wanyama zisizochemshwa au kupikwa kama maziwa au nyama zinaweza kusababisha maambukizi ya kifua kikuu ya matumbo ikiwa ni matokeo ya uhamasishaji wa ugojwa kutoka kwa wanyama kwenda kwa binadamu?
□ Ndio □ Hapana

41. Je ugonjwa wa kifua kikuu unaweza kutibika kwa dawa?
□ Ndio □ Hapana □ Sina uhakika

42. Je unajua inachukua muda gani kutibu kifua kikuu?
□ Ndio □ Hapana
Kama ndio, unaweza kukadiria muda wa matibabu? ______________________

43. Je watu wa jumuiya yako wanahusianisha ugonjwa wa kifua kikuu na virus vya UKIMWI?
□ Ndio □ Hapana □ Sina uhakika
Kama ndiyo kwa nini wanahusanisha kifua kikuu na virusi vya UKIMWI?
____

44. Kuna kitu chochote ambacho kingeweza kuleta urahisi wa watu wenye kifua kikuu kupata matibabu, sio kwenye kliniki hii tu, lakini kwenye vituo vingine vya afya?
□ Ndio □ Hapana □ Sina uhakika
Kama ndiyo, kitu gani kingeweza kufanyika? ____

45. Wakati ulivyotambua ya kwamba unaweza kuwa na maambukizi ya ugonjwa wa kifua kikuu ulipata matatizo yoyote kuamua kutafuta matibabu ya afya? Kama ndiyo, ni aina gani ya matatizo? ____
____
____

46. Ni hofu gani waliyonayo wengine kuhusu kifua kikuu ambayo inawazuia wasitafute ushauri wa kimatibabu?
____

47. Kama ulimwona mganga wa jadi kabla ya kutafuta huduma za matibabu za kisasa, ni saaababu zipi za mwanzo zilizopelekea umtumie mganga wa jadi kwanza?

GHARAMA KWA MGONJWA NA KAYA YAKE. Makisio ya kiwango cha mapato cha mgonjwa

48. Inachukua muda gani kwenda kwenye kituo cha huduma za afya kilicho karibu nawe?
□ Pungufu ya dakika 30 □ Kati ya dakika 30 na saa moja □ Zaidi ya saa moja

49. Ni umbali gani uliopo kati ya nyumbani kwako na hospitali hii (katika kilomita) ____________

50. Inachukua muda gani (kwa wastani) kwenye kituo hiki cha huduma za afya kusubiri kumuona daktari na kisha kurudi nyumbani au kazini? Saa___________________

51. Ulifikaje kwenye kituo hiki cha huduma za afya?
□ Nilitembea □ BaiskeliBicycle □ Pikipiki □ Gari binafsi □ Dala dala

52. Kama inakubidi kutumia daladala inagharimu shilingi ngapi (kwa wastani) kuja kwenye? kliniki ________________TZS.

53. Je, kawaida yako unakazi maalum za nymbani za kufanya kabla ya kuja kliniki?
□ Ndio □ Hapana □ Sina uhakika
Kama ndio ni taratibu zipi?__________________________

54. Je ni aina ipi kuu ya kazi unayojishughulisha nayo kwa kipindi cha miezi 12 iliyopita?
□ Mwajiriwa serikalini
□ Mwajiriwa sekta binafsi
□ Mwajiriwa shirika lisilo la kiserikali
□ Najiajiri, biashara na wafanyakazi
□ Najiajiri, biashara bila wa fanyakazi
□ Najiajiri (mfanyi biashara), mkulima/mvuvi
□ Sijaajiriwa
□ Nimestaafu
□ Mwanafunzi
□ Mlemavu/ mgonjwa
□ Mama wa nyumbani
□ Mengineyo_________________

55. Kipi ni chanzo kikuu cha mapato ya kaya yako?
□ Uzalishaji wa mazao
□ Ufugaji
□ Uvuvi
□ Uwindaji/ ufugaji nyuki
□ Ufugaji kuku
□ Kibarua mashambani
□ Shughuli nyingine za kilimo
□ Mshahara serikalini
□ Mshahara kwa watu binafsi
□ Kipato kwa njia ya riba
□ Kiinua mgongo
□ Kukodisha vitu
□ Mapato ya kujiajiri
□ Vyanzo vingine, vitaje ____________________________

56. Katika kipindi cha miezi 12 iliyopita ni aina gani za shughuli ulizokuwa unajishughulisha nazo wewe na wana kaya wenzako? (Shughuli za kuzalisha kipato tu)?
____
____
____

57. Kiasi gani ulicho (JINA) pata (fedha) kutokana na shughuli zilizo ainishwa katika kipindi cha miezi 12 iliyopita? Hii isihusishe tu mshahara au fedha taslim bali pia thamani ya bidhaa zilizozalishwa au kufanyiwa biashara kwa bidhaa nyingine au kwa za huduma.____

58. Je umepunguza uwezo wako wa utendaji kazi kutokana na ugonjwa wako wa sasa?
□ Ndio nimeacha kufanya kazi kabisa □ Ndio ninafanya kazi lakini kwa kiwango cha chini
□ Ninafanya kazi kama kawaida

59. Kama umepunguza kiwango cha kufanya kazi unakielezaje kiwango chako hicho capacity?
____

60. Ni lini uliacha kufanya kazi au kufanya kazi kwa kiwango cha chini?
____

61. Kuna mtu wa nyumbani aliyeacha kufanya kazi au kupunguza kiwango cha kufanya kazi kwa sababu ya ugonjwa wako?
 Ndio Hapana
Kama ndio, ni kwa muda gani? Siku _______________
Kama ndio, uwezo wa kufanya kazi ulipungua kwa kiasi gani? _______________________

62. Je wewe au mtu wa nyumbani ume/aliye poteza ajira/kipato kwasababu ya ugonjwa wako?
 Ndio Hapana Sina uhakika
Kama ndiyo, kiasi gani?____________________

63. Una miliki nyumba?
 Ndio Nyumba ya kupanga Ninaaishi na ndugu / marafiki Sina makazi

64. Ni watu wangapi wanaoishi katika nyumbani yako: Idadi ya watu _________________
Wangapi: Wanaume: _______ Wanawake:_______ Wazee:_______ Watoto (kati 0-10):_______
Watoto (kati 11-18): _______

65. Nini chanzo kikuu cha maji ya kunywa kwa wanafamilia wako?
 Maji ya Bomba 1 = ya ndani ya nyumba, 2= bomba nje, 3=Bomba ya umma, 4= Bomba ya Jirani
 Maji kutoka kisima cha wazi
 Maji kutoka kisima kilichofunikwa au kuchimba chini
 Maji yanayotiririka 1= chemchem, 2= mtoni/ mfereji 3= Dimbwi/Ziwa, 4= Bwawa
 Maji ya Mvua
 Maji ya kwenye lori la maji
 Maji ya kununua
 Maji ya chupa
 Vyanzo vingine vitaje ________________________

66. Ni aina gani ya huduma ya vyoo wanafamilia wako wanatumia?
 Choo cha maji Choo cha Shimo 1-Choo cha shimo cha kawaida, 2-Choo cha shimo chenye kuingiza hewa
 Hakuna huduma ya choo/kichakani/shambani Huduma nyingine, tafadhali taja ______________

67. Mnashirikiana huduma hizi na familia nyingine?
 Ndio Hapana

68. Je, nyumba yako ina?
 Umeme Taa za Kandili Radio
 TV Simu Za Mezani/ Mkononi Pasi za Umeme/ Mkaa
 Friji

69. Nini chanzo kikuu cha nishati ya mwanga katika nyumba yako?
 Umeme wa waya Nishati ya jua Gesi
 Taa za kandili Koroboi Kuni
 Mishumaa Vyanzo vingine vitaje _________________________

70. Ni vifaa gani vikuu vilivyotumika katika ujenzi wa kuta za nyumba yako au nyumba unayoishi?
 Majani/ nyasi Nguzo na matope Matofali ya saruji
 Matofali ya kuchoma Mbao Mawe
 Taja vifaa vingine _________________________

71. Ni aina gani ya vifaa vilivyotumika kuezeka nyumba yako au nyumba unayoishi?
☐ Nyasi/majani/ matope ☐ Mabati ☐ Vigae ☐ Saruji ☐ Bati za Asbesto
☐ Vifaa zinginezo, eleza _______________

72. Je, kuna mtu wa nyumbani yoyote anayemiliki
☐ Baiskeli ☐ Pikipiki/bajaji ☐ Gari ☐ Akaunti ya Benki

73. Je, kuna ekari ngapi za ardhi zinazomilikiwa na wanafamilia na ambazo zinatumika kwa ajili ya kilimo na ufugaji?
☐ Ardhi inayofaa kwa kilimo ekari_____ ☐ Ardhi inayofaa kwa ufugaji ekari______

74. Wanafamilia wako kwa kawaida wanapata milo mingapi kwa siku?
Milo_________________
