# Supplementary material for: MPT64 antigen detection test improves routine diagnosis of extrapulmonary tuberculosis in a low-resource setting: A study from the tertiary care hospital in Zanzibar
Source: PLoS One. 2018 May 9;13(5):e0196723. doi: 10.1371/journal.pone.0196723 (PMC5942825; doi:10.1371/journal.pone.0196723)
Supplement: S4 Text — (PDF) [file pone.0196723.s005.pdf]

S4 Text. Study questionnaire, Swahili version (patients < 18 years).

DODOSO – WATOTO (MIAKA < 18)

Tarehe:

Mshauri Bingwa:

Hospitali:

Idara: ☐ Idara ya wagonjwa wa Nje ☐ Idara ya wagonjwa wa Ndani

☐ Taarifa ridhaa ya maandishi ☐ Taarifa ridhaa ya mdomo

UTAMBULISHO WA MGONJWA:

Namba ya uchunguzi ____________________

Umri miaka _________

Jinsia: ☐ ME ☐ KE

Mhusika: ☐ Mgonjwa ☐ Mzazi ☐ Wengineo, tafadhali taja __________________________

Anwani: Mkoa _____________________ Kijiji _________________

1. Anaishi na:

☐ Mzazi mmoja (mama /baba) ☐ Wazazi wote ☐ Ndugu ☐ Yatima

☐ Wengine, tafadhali taja _______________________

2. Elimu/Uangalizi wa mchana

☐ Uangalizi nyumbani ☐ Vidudu ☐ Shule ya msingi

☐ Kati ya kidato cha I – IV ☐ Kidato cha IV – VI ☐ Nyinginezo (tafadhali taja)_________

3. Dini Madhehebu

☐ Muislam ☐ Mkristo ☐ Nyingineyo tafadhali taja _______________________

HISTORIA YA MATIBABU BINAFSI NA SIKU ZA NYUMA

4. Matumizi ya tumbaku (kijana) ☐ Ndio ☐ Hapana ____wiki/miezi/miaka

5. Uvutaji (kijana) ☐ Ndio ☐ Hapana ____wiki/miezi/miaka

6. Utumiaji wa pombe (kijana) ☐ Ndio ☐ Hapana ____wiki/miezi/miaka

7. Magonjwa husishwa

Pumu: ☐ Ndio ☐ Hapana

Kuharisha sugu: ☐ Ndio ☐ Hapana

Ugonjwa wa figo: ☐ Ndio ☐ Hapana

Ugonjwa wa ini: ☐ Ndio ☐ Hapana

Kisukari: ☐ Ndio ☐ Hapana

Ugonjwa wa moyo: ☐ Ndio ☐ Hapana

Mengineyo: ☐ Ndio ☐ Hapana

Eleza mengineyo:__________________________________

____

8. Dawa: *Tafadhali andika aina ya dawa alizo/ anazo tumia*

____

HISTORIA YA UGONJWA WA KIFUA KIKUU

9. Je, mtoto amewahi kukaribiana au kuwa na mtu anayejulikana kuwa na Kifua Kikuu?
 Ndio Hapana
Kama ndio, Ni nani / ama kina nani walikaribiana na mtoto?

____

Kama ndio, Lini mtoto alikaribiana na huyu/hawa watu?

____

10. Je, mtoto amewahi kuugua kifua kikuu?
 Ndio Hapana

11. Je, mtoto amewahi kuugua kifua kikuu nje ya mapafu?
 Ndio Hapana

12. Je, mtoto amewahi kutibiwa kifua kikuu?
 Ndio Hapana

13. Kama amewahi kutibiwa kifua kikuu, nini kilikuwa matokeo ya Matibabu?
 Alipona
 Matibabu yalikamilika
 Matibabu yalikatizwa

14. Kama alitibiwa, Ni lini mara ya mwisho alikamilisha matibabu yoyote ya kifua kikuu?

____

TABIA YA UTAFUTAJI WA MATIBABU

Tabia ya utafutaji matibabu kwa wagonjwa wa kifua kikuu

Tafadhali mfahamishe/ mkumbushe mgonjwa/ mhusika kwamba utafiti huu ni wa siri

15. Tafadhali uliza kama mgonjwa ana uzoefu wowote wa dalili zifuatazo

Dalili za ujumla

Homa: ☐ Ndio ☐ Hapana ____wiki/miezi

Una aina gani ya Homa?

☐ Kiwango cha Juu ☐ Kiwango cha Chini

Je, ni wakati gani mtoto anakuwa na homa?

☐ Asubuhi ☐ Mchana ☐ Jioni ☐ Usiku ☐ Siku nzima

Kushindwa kuongezeka uzito: ☐ Ndio ☐ Hapana ____wiki/miezi

Kupungua uzito ☐ Ndio ☐ Hapana ____wiki/miezi

Ukosefu wa Hamu ya Chakula: ☐ Ndio ☐ Hapana ____wiki/miezi

Utoaji Jasho Usiku: ☐ Ndio ☐ Hapana ____wiki/miezi

Uchovu: ☐ Ndio ☐ Hapana ____wiki/miezi

Udhaifu wa Mwili: ☐ Ndio ☐ Hapana ____wiki/miezi

Mafua ya mara kwa mara: ☐ Ndio ☐ Hapana ____wiki/miezi

Uvimbe shingoni: ☐ Ndio ☐ Hapana ____wiki/miezi

Mengineyo: ☐ Ndio ☐ Hapana ____wiki/miezi

Dalili kwenye ya njia ya hewa

Kukohoa: ☐ Ndio ☐ Hapana ____wiki/miezi

Makohozi: ☐ Ndio ☐ Hapana ____wiki/miezi

Kikohozi chenye makohozi: ☐ Ndio ☐ Hapana ____wiki/miezi

Kukohoa damu: ☐ Ndio ☐ Hapana ____wiki/miezi

Maumivu ya kifua: ☐ Ndio ☐ Hapana ____wiki/miezi

Kushindwa kupumua: ☐ Ndio ☐ Hapana ____wiki/miezi

Dalili kwenye ya njia ya chakula

Kuvimba/Uvimbe tumboni: ☐ Ndio ☐ Hapana ____wiki/miezi

Tumbo kujaa: ☐ Ndio ☐ Hapana ____wiki/miez

Kutapika: ☐ Ndio ☐ Hapana ____wiki/miezi

Kuharisha sugu: ☐ Ndio ☐ Hapana ____wiki/miezi

Mengineyo: ☐ Ndio ☐ Hapana ____wiki/miezi

Elezea dalili nyinginezo: ____

____

\

Dalili kwenye mfumo wa fahamu

Maumivu ya Kichwa: ☐ Ndio ☐ Hapana ____wiki/miezi

Kuhangaika/kuwa na hasira: ☐ Ndio ☐ Hapana ____wiki/miezi

Macho kuogopa mwanga: ☐ Ndio ☐ Hapana ____wiki/miezi

Kutapika: ☐ Ndio ☐ Hapana ____wiki/miezi

Kizunguzungu: ☐ Ndio ☐ Hapana ____wiki/miezi

Maruweruwe: ☐ Ndio ☐ Hapana ____wiki/miezi

Udhaifu wa mwili/miguu na mikono kufa ganzi:

☐ Ndio ☐ Hapana ____wiki/miezi

Matatizo ya macho: ☐ Ndio ☐ Hapana ____wiki/miezi

Mengineyo: □ Ndio □ Hapana ____wiki/miezi
Eleza dalili nyinginezo: ______________________________
____
____

16. Ni dalili zipi za ugonjwa ambazo zilikufanya utafute matibabu ya mwanzo kwa mwanao?
□ Kikohozi cha muda mrefu □ Kukohoa damu □ Kushindwa kupumua
□ Maumivu ya kifua □ Homa □ Kupungua uzito
□ Udhaifu wa mwili/ Uchovu □ Kukosa hamu ya chakula □ Kutokwa jasho usiku
□ Maumivu ya mifupa □ Kuvimba tezi □ Kuharisha
□ Maumivu ya tumbo □ Mengineyo (Taja) dalili nyingine _________________

17. Ni lini wewe au mwanao uli/ ali ziona dalili kwa mara ya kwanza?
____

18. Je, Ulimpatia mtoto matibabu yoyote binafsi kabla ya kutafuta huduma ya matibabu ya Afya
□ Ndio □ Hapana

19. Lini kwa mara ya kwanza ulitafuta ushauri wa kimatibabu kwa mtoto baada ya kugundua dalili?
□ Leo □ Kati ya siku 1-6 □ kati ya wiki 1 – 4 □ kati ya wiki 5-8 □ zaidi ya wiki 8

20. Sehemu ngapi tofauti ulikwenda kutafuta msaada wa ajili ya dalili za sasa za ugonjwa wa mtoto? Sehemu________________

21. Ni mara ngapi umepeleka mtoto kwenye huduma za afya akiwa na dalili hizi za ugonjwa?
□ Mara ya kwanza □ Mara mbili □ Mara ya tatu
□ Zaidi ya mara tatu □ Sikumbuki

22. Sehemu ipi ya kwanza ulitafuta huduma ya afya kutokana na dalili za ugonjwa wa mtoto?
□ Hospitali ya Mkoa □ Hospitali ya Wilaya □ Kituo cha Afya
□ Zahanati □ Hospitali ya binafsi □ Mganga wa Jadi
□ Duka la Dawa □ Nyinginezo, tafadhali taja ________________________________

23. Je, mtoto alipata dawa yoyote kutoka hapo?
□ Ndio □ Hapana

24. Kama ndiyo, alipata dawa aina gani?
□ Antibakteria □ Dawa za kifua kikuu
□ Miti Shamba □ Nyinginezo taja ____

25. Je, dalili za ugonjwa wa mtoto zilipungua baada ya kutumia dawa?
□ Ndio □ Hapana

26. Mtoto aligundulika na ugonjwa gani? ____

27. Kulikuwa na uchunguzi wowote wa kitabibu uliofanyika katika kituo cha kwanza cha afya ulichotembelea?
□ Ndio □ Hapana

28. Ni aina gani ya vipimo?
□ Kipimo cha damu □ Kipimo cha mkojo □ kipimo cha kikohozi □ Kipimo vya mionzi
□ Vipimo vinginevyo, eleza _________________________________

29. Je, uliyarudisha majibu ya vipimo kwa daktari?
□ Ndio □ Hapana

30. Je, unaweza kukadiria gharama ulizotumia awali kwa kwenda vituo vya afya au kufanyiwa uchunguzi kuhusiana na ugonjwa wa mtoto wa sasa?
Usajili kumwona daktari ________________TZS
Kulazwa ________________ TZS
Dawa ________________TZS
Vipimo vya maabara/x-ray/CT _________________TZS
Usafiri _________________TZS

31. Nani aliyempa mtoto rufaa kuja kwenye kituo hiki cha huduma za afya?
□ Mimi mwenyewe □ Mganga wa Jadi □ Viongozi wa kidini
□ Wauza madawa □ Wahudumu wa Afya wa kijiji □ Kituo cha Afya cha Serikali
□ Zahanati ya serikali □ Hospitali ya serikali □ Zahanati Binafsi / Hospitali
□ Vituo vya afya vya wahisani/mashirika yasiyo ya kiserikali □ Mwanafamilia
□ Wengineo: ___________________

32. Je mtoto wako amepata chanjo stahiki kwa kufuata mpango wa taifa wa chanjo kwa watoto?
□ Ndio □ Hapana

33. Je, mtoto wako amepata chanjo ya kukinga kifua kikuu?
□ Ndio □ Hapana

34. Je, mtoto amewahi kufanyiwa kipimo cha VVU/UKIMWI?
□ Ndio □ Hapana

35. Nini yalikuwa matokeo ya kipimo cha VVU/UKIMWI?
□ Maambukizi □ Hakuna maambukizi □ Sijui □ Siwezi kusema hali yake ya maambukizi

36. Kabla ya leo, ulikuwa umewahi kusikia ugonjwa wa kifua kikuu?
□ Ndio □ Hapana

37.Unazijua dalili zozote za ugonjwa wa kifua kikuu?
□ Kikohozi Sugu □ Kutema damu □ Kushindwa kupumua
□ Maumivu ya kifua □ Homa □ Kupungua uzito
□ Kuchoka □ Kukosa hamu ya chakula
□ Nyinginezo tafadhali taja ______________________________ ***(Usihoji bali uliza dalili zaidi za ugongwa wa kifua kikuu)***

38. Unajua ni sehemu gani mwilini zinazoweza kuathiriwa na kifua kikuu?
____

39. Je, ugonjwa wa kifua kikuu unaweza kuambukiza kutoka kwa mtu mmoja kwenda kwa mwingine?
□ Ndio □ Hapana □ Sina uhakika

40. Je, kwenye nyumbani mwako mnakunywa maziwa yasiyochemshwa?
□ Ndio □ Hapana

41. Je, kwenye nyumbani mwako mnakula nyama mbichi?
□ Ndio □ Hapana

42. Je ulijua kwamba utumiaji / ulaji wa bidhaa za wanyama zisizochemshwa au kupikwa kama maziwa au nyama zinaweza kusababisha maambukizi ya kifua kikuu cha matumbo ikiwa ni matokeo ya uhamasishaji wa ugojwa kutoka kwa wanyama kwenda kwa binadamu?
☐ Ndio ☐ Hapana

43. Je, ugonjwa wa kifua kikuu unaweza kutibika kwa dawa?
☐ Ndio ☐ Hapana ☐ Sina uhakika

44. Je, unajua inachukua muda gani kutibu kifua kikuu?
☐ Ndio ☐ Hapana
Kama ndio, unaweza kukadiria muda wa matibabu? ______________________

45. Je, watu wa jumuiya yako wanahusianisha ugonjwa wa kifua kikuu na virus vya UKIMWI?
☐ Ndio ☐ Hapana ☐ Sina uhakika
Kama ndiyo kwa nini wanahusanisha kifua kikuu na virusi vya UKIMWI?
____

46. Kuna kitu hochote ambacho kingeweza kuleta urahisi wa watu wenye kifua kikuu kupata matibabu, sio kwenye kliniki hii tu, lakini kwenye vituo vingine vya afya?
☐ Ndio ☐ Hapana ☐ Sina uhakika
Kama ndiyo, kitu gani kingeweza kufanyika? ____

47. Wakati ulivyotambua ya kwamba mtoto anaweka kuwa na maambukizi ya ugonjwa wa kifua kikuu ulipata matatizo yoyote kuamua kutafuta matibabu ya afya? Kama ndiyo, ni aina gani ya matatizo? ____
____
____

48. Ni hofu gani waliyonayo wengine kuhusu kifua kikuu ambayo inawazuia wasitafute ushauri wa kimatibabu?
____

49. Kama ulimwona mganga wa jadi kabla ya kutafuta huduma za matibabu za kisasa, ni saaababu zipi za mwanzo zilizopelekea umtumie mganga wa jadi kwanza?

GHARAMA KWA MGONJWA NA KAYA YAKE. Makisio ya kiwango cha mapato cha mgonjwa

50. Inachukua muda gani kwenda kwenye kituo cha huduma za afya kilicho karibu nawe?
 Pungufu ya dakika 30 Kati ya dakika 30 na saa moja Zaidi ya saa moja

51. Ni umbali gani uliopo kati ya nyumbani kwako na hospitali hii (katika kilomita) ____________

52. Inachukua muda gani (kwa wastani) kwenye kituo hiki cha huduma za afya kusubiri mtoto aonane na daktari na kisha kurudi nyumbani au kazini? Saa___________________

53. Ulifikaje kwenye kituo hiki cha huduma za afya?
 Nilitembea BaiskeliBicycle Pikipiki Gari binafsi Dala dala

54. Kama inakubidi kutumia daladala inagharimu shilingi ngapi (kwa wastani) kuja kwenye? kliniki ________________TZS.

55. Je, kawaida yako unakazi maalum za nymbani za kufanya kabla ya kuja kliniki??
 Ndio Hapana Sina uhakika
Kama ndio ni taratibu zipi?__________________________

56 Kipi ni chanzo kikuu cha mapato ya kaya yako?
 Uzalishaji wa mazao Ufugaji Uvuvi
 Uwindaji/ ufugaji nyuki Ufugaji kuku Kibarua mashambani
 Shughuli nyingine za kilimo Mshahara serikalini Mshahara kwa watu binafsi
 Kipato kwa njia ya riba Kiinua mgongo
 Kukodisha vitu Mapato ya kujiajiri
 Vyanzo vingine, vitaje ____________________________

57. Katika kipindi cha miezi 12 iliyopita ni aina gani za shughuli ulizokuwa unajishughulisha nazo wewe na wana kaya wenzako? (Shughuli za kuzalisha kipato tu)?
____
____

58. Kiasi gani ulicho (JINA) pata (fedha) kutokana na shughuli zilizo ainishwa katika kipindi cha miezi 12 iliyopita? Hii isihusishe tu mshahara au fedha taslim bali pia thamani ya bidhaa zilizozalishwa au kufanyiwa biashara kwa bidhaa nyingine au kwa za huduma.
____

59. Kuna mtu wa nyumbani aliyeacha kufanya kazi au kupunguza kiwango cha kufanya kazi kwa sababu ya ugonjwa wa mtoto?
 Ndio Hapana
Kama ndio, ni kwa muda gani? Siku _______________
Kama ndio, uwezo wa kufanya kazi ulipungua kwa kiasi gani? ________________________

60. Je wewe au mtu wa nyumbani ume/aliye poteza ajira/kipato kwasababu ya ugonjwa wa mtoto?
 Ndio Hapana Sina uhakika
Kama ndiyo, kiasi gani?____________________

61. Una miliki nyumba?
 Ndio Nyumba ya kupanga Ninaaishi na ndugu / marafiki Sina makazi

62. Ni watu wangapi wanaoishi katika nyumbani yako: Idadi ya watu _________________

Wangapi: Wanaume: _______ Wanawake:_______ Wazee:_______ Watoto (kati 0-10):_______ Watoto (kati 11-18): _______

63. Je, mtoto ana ndugu wangapi wa kuzaliwa nao? Ndugu_____________

64. Nini chanzo kikuu cha maji ya kunywa kwa wanafamilia wako?
 Maji ya Bomba 1 = ya ndani ya nyumba, 2= bomba nje, 3=Bomba ya umma, 4= Bomba ya Jirani
 Maji kutoka kisima cha wazi
 Maji kutoka kisima kilichofunikwa au kuchimba chini
 Maji yanayotiririka 1= chemchem, 2= mtoni/ mfereji 3= Dimbwi/Ziwa, 4= Bwawa
 Maji ya Mvua
 Maji ya kwenye lori la maji
 Maji ya kununua
 Maji ya chupa
 Vyanzo vingine vitaje _________________________

65. Ni aina gani ya huduma ya vyoo wanafamilia wako wanatumia?
 Choo cha maji Choo cha Shimo 1-Choo cha shimo cha kawaida, 2-Choo cha shimo chenye kuingiza hewa
 Hakuna huduma ya choo/kichakani/shambani Huduma nyingine, tafadhali taja _______________

66. Mnashirikiana huduma hizi na familia nyingine?
 Ndio Hapana

67. Je, nyumba yako ina?
 Umeme Taa za Kandili Radio
 TV Simu Za Mezani/ Mkononi Pasi za Umeme/ Mkaa
 Friji

68. Nini chanzo kikuu cha nishati ya mwanga katika kaya yako?
 Umeme wa waya Nishati ya jua Gesi
 Taa za kandili Koroboi Kuni
 Mishumaa Vyanzo vingine vitaje __________________________

69. Ni vifaa gani vikuu vilivyotumika katika ujenzi wa kuta za nyumba yako au nyumba unayoishi?
 Majani/ nyasi Nguzo na matope Matofali ya saruji
 Matofali ya kuchoma Mbao Mawe
 Taja vifaa vingine __________________________

70. Ni aina gani ya vifaa vilivyotumika kuezeka nyumba yako au nyumba unayoishi?
 Nyasi/majani/ matope Mabati Vigae Saruji Bati za Asbesto
 Vifaa zinginezo, eleza ________________

71. Je, kuna mtu wa nyumbani yoyote anayemiliki
 Baiskeli Pikipiki/bajaji Gari Akaunti ya Benki

72. Je, kuna ekari ngapi za ardhi zinazomilikiwa na wanafamilia na ambazo zinatumika kwa ajili ya kilimo na ufugaji?
 Ardhi inayofaa kwa kilimo ekari_____ Ardhi inayofaa kwa ufugaji ekari______

73. Wanafamilia wako kwa kawaida wanapata milo mingapi kwa siku?
Milo_________________
